# Metabolic and evolutionary insights into the closely-related species *Streptomyces coelicolor *and *Streptomyces lividans *deduced from high-resolution comparative genomic hybridization

**DOI:** 10.1186/1471-2164-11-682

**Published:** 2010-12-01

**Authors:** Richard A Lewis, Emma Laing, Nicholas Allenby, Giselda Bucca, Volker Brenner, Marcus Harrison, Andrzej M Kierzek, Colin P Smith

**Affiliations:** 1 Microbial Sciences Division, Faculty of Health and Medical Sciences, University of Surrey, Guildford, UK; 2 Oxford Gene Technology Ltd, Begbroke Business Park, Sandy Lane, Yarnton, Oxford, UK; 3 Mediwatch Biomedical Ltd, Colworth House, Sharnbrook, Bedfordshire, UK

## Abstract

**Background:**

Whilst being closely related to the model actinomycete *Streptomyces coelicolor *A3(2), *S. lividans *66 differs from it in several significant and phenotypically observable ways, including antibiotic production. Previous comparative gene hybridization studies investigating such differences have used low-density (one probe per gene) PCR-based spotted arrays. Here we use new experimentally optimised 104,000 × 60-mer probe arrays to characterize in detail the genomic differences between wild-type *S. lividans *66, a derivative industrial strain, TK24, and *S. coelicolor *M145.

**Results:**

The high coverage and specificity (detection of three nucleotide differences) of the new microarrays used has highlighted the macroscopic genomic differences between two *S. lividans *strains and *S. coelicolor*. In a series of case studies we have validated the microarray and have identified subtle changes in genomic structure which occur in the Asp-activating adenylation domains of CDA non-ribosomal peptide synthetase genes which provides evidence of gene shuffling between these domains. We also identify single nucleotide sequence inter-species differences which exist in the actinorhodin biosynthetic gene cluster. As the glyoxylate bypass is non-functional in both *S. lividans *strains due to the absence of the gene encoding isocitrate lyase it is likely that the ethylmalonyl-CoA pathway functions as the alternative mechanism for the assimilation of C_2 _compounds.

**Conclusions:**

This study provides evidence for widespread genetic recombination, rather than it being focussed at 'hotspots', suggesting that the previously proposed 'archipelago model' of genomic differences between *S. coelicolor *and *S. lividans *is unduly simplistic. The two *S. lividans *strains investigated differ considerably in genetic complement, with TK24 lacking 175 more genes than its wild-type parent when compared to *S. coelicolor*. Additionally, we confirm the presence of *bldB *in *S. lividans *and deduce that *S. lividans *66 and TK24, both deficient in the glyoxylate bypass, possess an alternative metabolic mechanism for the assimilation of C_2 _compounds. Given that streptomycetes generally display high genetic instability it is envisaged that these high-density arrays will find application for rapid assessment of genome content (particularly amplifications/deletions) in mutational studies of *S. coelicolor *and related species.

## Background

Bacteria of the Gram-positive genus *Streptomyces *are of great scientific and economic importance. Streptomycetes are saprophytic, aerobic, soil dwelling bacteria which undergo complex differentiation to form mycelium, aerial hyphae and spores, and produce a diverse array of secondary metabolites, such as antibiotics and bioactive compounds [1]. There are a number of published streptomycete genomes including *Streptomyces griseus *[2], *Streptomyces avermitilis *[3] and *Streptomyces coelicolor *[4]. The genomes of the sequenced streptomycetes consist of large (6-12 Mb) linear chromosomes with high (72-74%) G+C content [4]. The chromosomes show synteny to each other in the central 'core', a region thought to contain conserved and highly expressed genes, and also contain two flanking 'arm' regions, proposed regions of high horizontal gene transfer (HGT) with high recombination rates [5,6]. The large sizes of streptomycete genomes are thought to be related to the diversity of environmental niches which *Streptomyces *encounter.

*S. lividans *is known to be closely related to *S. coelicolor *A3(2) and has a virtually identical (99.7%) 16S rDNA sequence [7,8]. Both species are members of the *S. violaceoruber *clade in 16S rDNA phylogenetic trees [9-11]. The similarities in basic genomic structure and genetic organisation of the *S. coelicolor *and *S. lividans *chromosomes have long been known [12]. However, it is clear that many genomic differences are present, e.g. *S. lividans *66 possesses a 93 kb "genomic island" in its chromosome, relative to that of *S. coelicolor *A3(2) [13,14], and does not contain the same fertility plasmids as *S. coelicolor*, A3(2) instead harbouring the plasmids SLP2 [15] and SLP3 [16]. These, and other genetic differences, are reflected in phenotypic differences. For example, *S. lividans *differs from *S. coelicolor *in that its DNA is degraded during electrophoresis in buffers containing traces of ferrous iron. This phenomenon is due to a DNA phosphorothioate modification conferred by a cluster of five *dnd *genes which are only present in the *S.lividans *genome in the 93 kb island [13,17-19]. Although, *S. lividans *exhibits most of the secondary metabolic capability that *S. coelicolor *possesses it does not produce antibiotics to the same extent, for reasons which are unclear. However, a number of studies have shown that antibiotic gene clusters can be "awakened" in *S. lividans *through the over-expression of regulatory factors such as AfsS and ActII-ORF4 [20,21].

*S. lividans *is one of the most commonly used *Streptomyces *hosts for DNA cloning [22,23] and heterologous protein production [24,25]. It has several features that make it a suitable host for efficient recombinant protein expression, including the absence of a methylation-dependent restriction system [26] which recognizes and degrades methylated DNA isolated from commonly used *Escherichia coli *strains [27,28]. Additionally, *S. lividans *has very low endogenous extracellular proteolytic activity when compared to other *Streptomyces *species, leading to higher product recovery [29]. Moreover, in comparison with *E. coli*, *S. lividans *is a better host for eukaryotic recombinant protein production because the recombinant proteins produced in *S. lividans *tend to have higher levels of solubility, therefore avoiding the problem of inclusion body formation.

For streptomycetes for which no genome sequence is available, comparative genomic hybridization (CGH), through the use of microarrays, provides a useful tool for the comparison of genetic content between strains. While microarrays cannot detect chromosomal rearrangements or single nucleotide polymorphisms, oligonucleotide based microarrays can be hybridized using stringent conditions which enable the detection of small numbers of bases changes, and can be a powerful tool in detecting gene duplication, horizontal gene transfer (HGT) and gene loss/divergence. A previous comparative study of *S. lividans *TK21 against *S. coelicolor *M145 has been conducted by Jayapal and co-workers [30]. However, because spotted PCR product based arrays were used the findings were limited to identification of large-scale differences, (PCR product probes being more tolerant to nucleotide changes compared to oligonucleotide probes) between gene coding regions. In the present study the genomic differences between the sequenced *S. coelicolor *M145 strain, (a prototrophic, plasmid-free derivative of the wild-type A3(2) strain) and two *S. lividans *strains: *S. lividans *66 (wild-type), and its plasmid free derivative strain *S. lividans *TK24, have been determined using our novel high-density *S. coelicolor *microarrays. Our aim was to determine whether high-resolution CGH could identify small insertions/deletions (indels). It was anticipated that this study would serve not only to experimentally validate the microarray platform, but also shed light on the genotypic differences between the two streptomycete species, and also between the two strains of *S. lividans*.

## Results and Discussion

### Production of high density 104K × 60-mer *S. coelicolor *DNA microarray

Due to the high G+C content of *S. coelicolor *(ca. 72%) it was necessary to experimentally test specificity of a large set of probes in order to select a validated subset for comprehensive coverage of the genome. The resulting 104K microarray comprises almost 104,000 unique 60-mers (see Material & Methods) with an average spacing of 30 nucleotides. The 104K array is designed for CGH, ChIP-Chip and high resolution transcriptome analysis, covering all known genes and all intergenic regions; both strands of the genome are represented for the latter.

### High resolution comparative genomics

**Figure 1:**
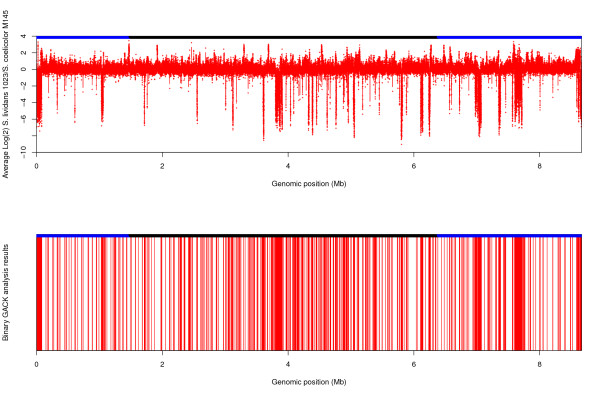
**Chromosome plot of *S. coelicolor *M145 versus *S. lividans 66***. **(A) **Log_2 _ratio of probe signals from *S. lividans *66/*S. coelicolor *M145 (*S. liv *66 */S. coel *M145). Low values imply absence or divergence of gene in *S. lividans *66. **(B) **Binary representation (as classified by GACK analysis) *of S. lividans *66 probe data, where a red line indicates absence and white presence. Black bars at the top of the figures denote the core [4] region of the chromosome; blue bars at the top of the figures denote the 'arm' regions of the chromosome [4].

The availability of the genome sequence of *S. coelicolor *allows the use of post-genomic technologies such as microarrays to explore the genomic content of other *Streptomyces *species. Here we have used the 104K array to examine the genomic differences between *S. coelicolor *M145 and *S. lividans *66 and its plasmid free derivative strain *S. lividans *TK24 using novel, ink-jet *in situ *synthesized (IJISS) high density microarrays. Thus, the averaged (across dye-swapped biological replicates) normalised log_2 _ratios, with M145 genomic DNA as the common denominator (reference sample), for each probe can be ordered by the genomic position as it appears in the annotated *S. coelicolor *genome sequence. Consequently, the differences between the two *S. lividans *strain chromosomes and the *S. coelicolor *M145 chromosome can be readily observed and compared (Figure 1A). Furthermore, by importing the data into GACK (Genome Analysis by Charles Kim), software built around an algorithm that selects a dynamic cut-off value based on the shape of the signal ratio distribution [31], a binary classification of presence/absence could be applied to generate a list of present/absent probes which is represented diagrammatically in Figure 1B.

*Prima facie*, we were confident that regions of difference identified by multiple contiguous probes indeed represented genuine phenomena and demonstrated the presence of substantial sequence differences between *S. coelicolor *and the *S. lividans *strains. However, initially our confidence in the validity of single probe absences was less. The microarray probe design and validation procedure [32] was such that only probes that exhibited a reduction in signal intensity (and thus represent a non-binding/absent event) following inclusion of a three nucleotide mismatch were selected. Therefore, to experimentally verify in a real world experimental situation whether the apparently missing isolated probes were in fact genuine absences, or false negatives, and whether the microarrays were operating according to specification, we selected several regions targeted by these single, isolated 'absent' probes for further analysis.

### Investigation of a single non-binding probe in the CDA biosynthetic gene cluster

**Figure 2:**
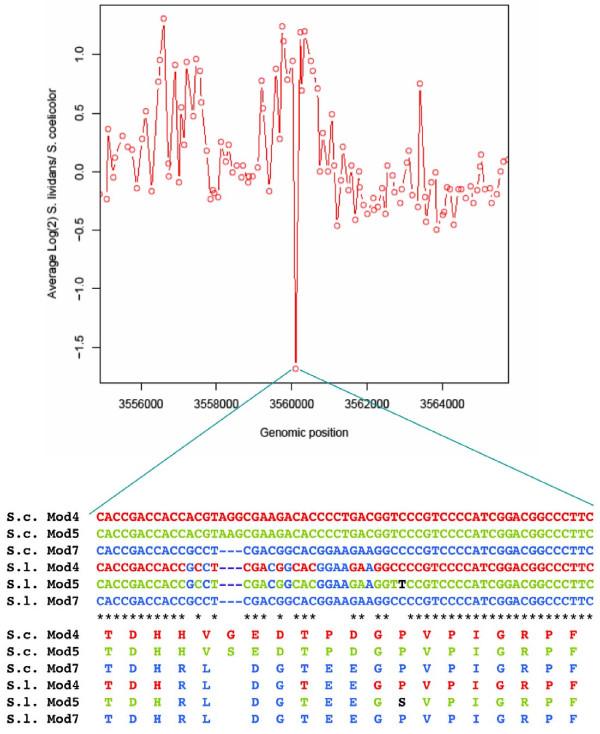
**Detection of a single divergent microarray probe in the *cda *gene cluster**. Upper panel: Log_2 _ratio of probe signals from *S. lividans *66/*S. coelicolor *M145. Lower panel: Sequences of *S. coelicolor *M145 (S.c) and *S. lividans *66 (S.l) corresponding to nucleotide coordinates 5524009-5524068 of the *S. coelicolor *M145 chromosome (Accession No. EMBL: AL645882.2).

Analysis of the results obtained using the 104K array design (see Materials and Methods) indicated the apparent absence/divergence in both *S. lividans *strains of a probe-binding sequence corresponding to nucleotides 3560106-3560166 of the *S. coelicolor *chromosome (Figure 2), *i.e*. in the adenylation domain of Module 5 (Asp) of CdaPSI of the calcium dependent antibiotic (CDA) biosynthetic cluster [33]. Also, significantly, absence of this probe-binding sequence suggested the absence of the corresponding sequence present in the adenylation domain of Module 4 (also an aspartate activating domain) in *S. lividans *66, as the sequence in this region in *S. coelicolor *is identical to that of Module 5. In order to verify that this was indeed the case, regions of the adenylation domains of *S. lividans *CdaPS Modules 4, 5 & 7 spanning the 'missing' probe were amplified by PCR using specific primer pairs whose sequences are based on the published *S. coelicolor *M145 genome sequence (Additional File 1). The sequences of the cloned PCR products (shown in Figure 2) show that the sequence of the *S. lividans *66 Module 5 and Module 4 differ from the *S. coelicolor *probe sequence by a three bp deletion which is flanked by regions containing eleven base mismatches. These results indicate that Modules 4 and 5 of *S. lividans *66 contain sequences which are identical to that of *S. lividans *66 and *S. coelicolor *Module 7 (another Asp Module) and are different from *S. coelicolor *Modules 4 & 5. It is likely that the sequence differences between the species derive from recombination events between the three Asp modules in *S. lividans *66 resulting in the Module 4 & 5 sequences being replaced by the corresponding sequence from Module 7. However, a close examination of longer sequences of Modules 4, 5 and 7 indicates that, relative to the *S. coelicolor *sequences, the three modules of *S. lividans *are 'shuffled' and each *S. lividans *module contains specific nucleotides/motifs characteristic of all three *S. coelicolor *Asp modules (Additional File 2). This data suggests a long history of recombination events between these three Asp modules, although due to the high levels of similarity between them, identification of cross-over sites has proved elusive. It is significant that the sequences encoding the active site residues which determine the specificity of modules 4, 5 & 7 for aspartate has been conserved [34] even though there has been recombination within the modules; this is consistent with the aspartate residues being essential for CDA activity as they are responsible for the binding of a Ca^2+ ^ion [35,36].

### Investigation of a single non-binding probe in the actinorhodin gene cluster

From a preliminary, trial array used in our array testing and optimization process (see Methods) we identified a non-binding probe corresponding to positions 5524009-5524068 of the *S. coelicolor *chromosome, *i.e*. a region located in between genes SCO5082 and SCO5083 of the actinorhodin biosynthetic cluster, which encode the *actII-1 *transcriptional regulator and *actII-2 *actinorhodin transporter proteins respectively. Sequence determination of a PCR product amplified using primers actdelL and actdelR (Additional File 1) spanning the corresponding region of the *S. lividans *66 chromosome generated the sequence shown in Additional File 3, which differs from the *S. coelicolor *probe sequence by seven nucleotides, including three consecutive nucleotide mismatches. It may be that these sequence differences, which are located in the divergent promoter region of SCO5082-3, are at least partly responsible for the differences in actinorhodin production between *S. coelicolor *and *S. lividans*, although it is clear that other factors are involved in actinorhodin production/regulation [[20,21]; see below].

The above results illustrate the sensitivity of the experimentally optimised 60-mer probe set in discriminating relatively small differences in nucleotide sequence.

### Establishment of the "region of difference" calling criterion for gene presence/absence

The above studies demonstrate the high specificity of our microarray design, indicating that single non-binding probes can represent genuine genomic differences between strains. In addition to providing detection of small sequence differences and microdissection of individual gene structure our results provide a macroscopic overview of genome content in *S. lividans*.

**Figure 3:**
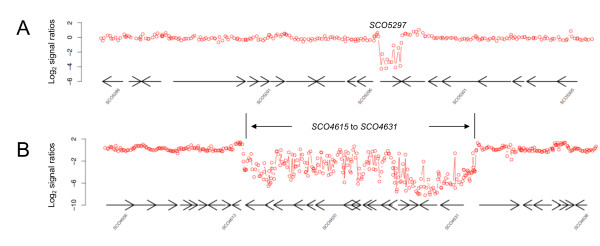
**"Regions of difference" within (A) the transposase gene SCO5297 and (B) Genomic Island 11 (Gi-11) (SCO4615 to SCO4631)**.

In a previous comparative study of *S. lividans *TK21 against *S. coelicolor *M145 Jayapal and co-workers [30] used a 7,579 probe spotted PCR array, where each gene is targeted by one probe. They used a criterion that three consecutive probe targeting genes need to be classified as divergent/absent before being assigned as absent. In analogy to their criterion, our presence/absence calling criterion is based on a relative loss of signal from a minimum of three consecutive 60-mer probes, which define a 'region of difference'. Figure 3 illustrates how the pattern of probe binding may be used to characterize 'regions of difference'. Genes are classified as 'absent' if their annotated translational start site (as in *Streptomyces *EMBL Accession No. EMBL: AL645882.2) is encompassed by a region satisfying the 'region of difference/absence' criterion. We believe that this analysis represents a conservative view of the number of absent/divergent genes, as, for example, it would assign 3" truncated non-functional genes as present. This situation is illustrated in Figure 3A: SCO5297 is not classified as absent/divergent despite it containing a 'region of difference' comprising 10 non-binding probes in both *S. lividans *strains as its translational start site is not encompassed within this region. The converse situation also occurs where our calling criterion classifies genes whose 5' terminus lies within a "region of difference" as "absent". For example, we classify SCO6832 (a methylmalonyl-CoA mutase) as being absent/divergent in *S. lividans *TK24. It is clear from the GACK classification of probe absences (see Additional Files 4 and 5) that SCO6832 is largely intact/present, as of the 18 probes targeting this gene all but three are present/non-divergent, *i.e*. only the first probe targeting the 5' terminus and the two probes targeting the 3' terminus do not bind. However, significantly, as a run of more than three consecutive non binding probes, comprising the first probe within the gene, extends into SCO6832 from upstream so encompassing the translational start site of the gene within a 'region of difference', the gene is classified as absent/divergent according to the microarray data. However, the results of our reciprocal BLAST search (see below) indicated that SCO6832 is present in *S. lividans *TK24 and diverges only from the *S. coelicolor *gene sequence at the 5' and 3' termini in a pattern consistent with that of the microarray probe-binding (see Additional File 6).

Genes classified as absent/divergent from both *S. lividans *66 and TK24, and uniquely from TK24 are given, respectively, in Additional Files 7, 8 and 9. The criterion used in this study represents a significant advance in terms of increase in resolution of interspecies genomic differences over the previous amplicon-based CGH study [30]. Furthermore, the present study identifies differences between the intergenic regions of *S. coelicolor *and *S. lividans *which have not been previously investigated.

**Figure 4:**
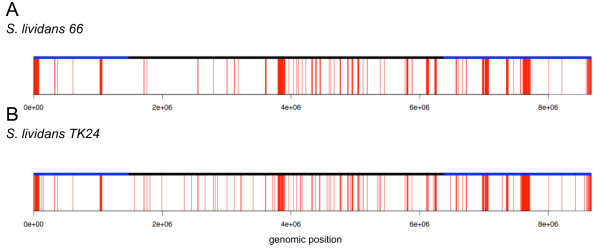
**Chromosome binary plots (as classified by GACK analysis) of *S. coelicolor *M145 versus *S. lividans***. **(A) ***S. lividans *66 probe data, **(B) ***S. lividans *TK24 probe data. A red line indicates absence and white presence.

From Figure 1 it is apparent that *S. lividans *66 shares the vast majority of its genome with *S. coelicolor *A3(2). We have identified 6,138 and 7,885 probe differences between *S. lividans *66 and *S. lividans *TK24 and *S. coelicolor *M145, respectively. These include 691 single probe absences and 179 double probe absences for *S. lividans *66 relative to *S. coelicolor *M145, and 436 single probe and 114 double probe absences for *S. lividans *TK24, relative to *S. coelicolor *M145, respectively. According to our calling criterion these probe absences do not contribute towards assigning genes as present/absent. When our calling criterion was applied to the remaining absent probes which are present in consecutive runs of three or more we identify 512 'regions of difference' between *S. lividans *66 and *S. coelicolor *M145, which encompass 444 absent/divergent genes, and 383 'regions of difference' between *S. lividans *TK24 and *S. coelicolor*, encompassing 619 absent/divergent genes. The distribution of these 'regions of difference' across the *S. lividans *66 and TK24 chromosomes is shown schematically in Figure 4 (*S. lividans *66, Figure 4A; *S*. *lividans *TK24, Figure 4B).

We note that there are 'regions of difference' which do not appear to correlate with absent/divergent protein coding genes. The absence/divergence of these regions may however prove to be significant as these regions may encompass promoter elements and RNA genes, including, for example at present unidentified/uncharacterized *cis*- or *trans*-encoded non-coding RNAs.

### Comparison of the *S. lividans *TK24 microarray results with the results of BLAST search

**Figure 5:**
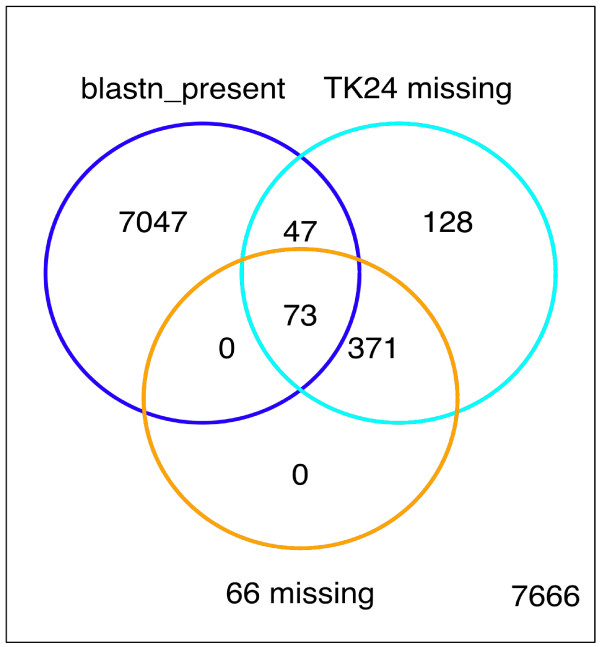
**Genes absent from *S. lividans *TK24**. Venn diagram illustrating numbers of absent genes, according to GACK analysis of the *S. lividans *TK24 microarray dataset and a reciprocal BLAST search of the Broad Institute S. *lividans *TK24 sequence database.

The Broad Institute (USA) are currently in the process of annotating the sequence of *S. lividans *TK24 and the sequence data are available for BLAST search *via *their website [37]. As a test of our calling criteria we performed a reciprocal BLAST search (see Methods) between the *S. coelicolor *M145 and *S. lividans *TK24 genes; the results are presented in Figure 5. Both the microarray and BLAST results find 498 genes to be absent/divergent in *S. lividans *TK24 relative to *S. coelicolor *M145. However, 120 genes are classified as absent/divergent from the microarray data but are present according to the BLAST results. One possible explanation for these results may be due to the difference in discrimination of the techniques employed. For families of very similar/identical genes the BLAST search may have identified absent/divergent genes as present through targeting their similar family members, whereas the microarray probes, which are designed to be specific for each gene, give a more accurate indication of presence/absence. In this regard it is worth noting that many of the genes classed as present/non divergent according to the reciprocal BLAST results (27 out of 120) are transposase genes which give multiple hits when 'BLASTed' against the *S. coelicolor *M145 genome; transposase genes that give smaller numbers of BLAST hits are comprised within the 498 genes identified as absent/divergent by both the microarray analysis and reciprocal BLAST search. An example illustrating the differences in specificity of the microarray probes and BLAST search is the case of SCO6833 (an isobutyryl-CoA mutase small subunit) which appears from the pattern of microarray probe-binding (see Additional Files 4 &5) to be absent/divergent from *S. lividans *TK24 but which the reciprocal BLAST search (see Additional File 6) classifies as present/non divergent.

From the annotated 7,824 protein encoding genes in the *S*. *coelicolor *genome, the BLAST analysis identifies 657 genes as missing and 7,167 as present in *S. lividans *TK24. From the array analysis of *S*. *lividans *TK24 versus *S*. *coelicolor *M145 we identify 619 genes as missing and 7,205 as present. 120 genes were found to be missing by the array analysis but found to be present by BLAST, whilst 159 genes were found to be present by the array analysis but missing by BLAST.

If we classify genes identified as present by the reciprocal BLAST as True Positives (TP) and the number of genes mis-identified (when compared to the reciprocal BLAST) by the array classed as False Negatives (FN) then the sensitivity can be calculated by TP/TP+FN. Thus the sensitivity of the arrays and calling method is 7047/7047+120: 98%. If we class genes identified as absent by the reciprocal BLAST as True Negatives (TN) and the number of genes mis-identified (when compared to the reciprocal BLAST) by the array classed as False Positives (FP) then the Specificity can be calculated by TN/TN+FP. Thus the specificity of the arrays and calling method is 498/498+159: 78%. The overall accuracy (TN+TP/TN+TP+FN+FP) of the arrays and calling method for CGH purposes is 96%.

### Macroscopic pattern of gene absence/divergence in *S. lividans *66 and TK24 and comparision to *S. lividans *TK21 and *S. coelicolor *M145

Previous studies [30] have categorized regions of difference based on size as either Genomic Islands (GI) (≥25 kb) or as smaller Genomic Islets (Gi), which although smaller than 25 kb contain at least three consecutive genes [30]. The use of this "archipelago" model is understandable given the technical limitations of earlier microarray technologies and the gene absence/presence calling criterion, which tended to bias the analysis towards identifying blocks of consecutive absent genes. Analysis of the *S. coelicolor *M145 genome sequence identified a series of regions designated as potentially recently laterally acquired [4] which are broadly consistent with clusters of genes identified by Jayapal and co-workers as absent/divergent. However, the increased resolution of the genomic differences between the species that the 104K microarray of the present study affords has allowed us to identify a more subtle and complicated pattern of differences, so the 'archipelago' model approach was not employed in our study.

**Figure 6:**
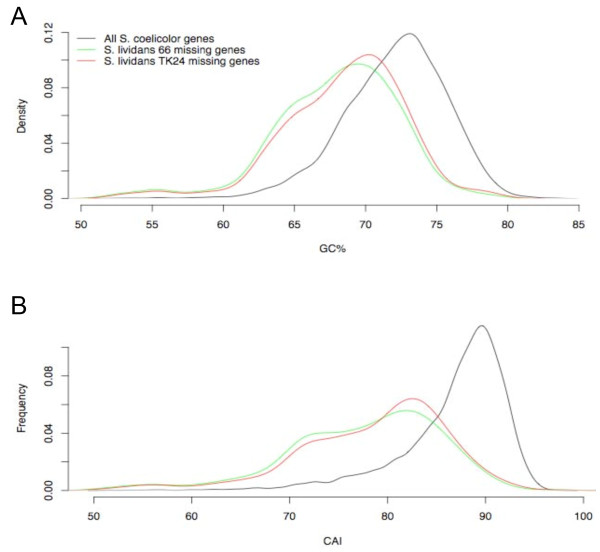
**%G+C content and Codon Adaptation Index distributions of all *S. coelicolor *genes compared to missing genes in *S. lividans *66 and TK24**. (**A**) G+C content (%) of protein-encoding nucleotide sequences, (**B**) Codon Adaptation Index (CAI) of protein-encoding nucleotide sequences.

It is clear from Figure 4 that the identified 'regions of difference' are not evenly distributed along the *S. lividans *66 and TK24 chromosomes. In common with previous studies [4,30] we note that certain regions containing tRNA genes and integrated copies of the *S. ambofaciens *plasmid pSAM2 homologues are hotspots for gene absence/divergence, due to recombinogenic activity. However, our results lead us to propose that rather than being strictly confined to these isolated areas, gene deletion/divergence occurs more widely throughout the *S. lividans *chromosome than previously thought. It appears that in both *S. lividans *strains, "regions of difference" and single and double absent probes are distributed throughout the genome, with a slight tendency to occur in the "core" region (Figures 1 and 4) as previously defined by [4]. Both *S. lividans *strains 66 and TK24 possess significant deletions at the chromosome termini, which is consistent with previous observations regarding *S. lividans *TK21 and *S. coelicolor *M145, and with other studies reported in the literature [6,38], reporting that the terminal regions of streptomycete genomes are more prone to deletion, duplication and recombination events. Evaluation of the % G+C content of the genes identified as absent/divergent in the present study indicates that they are unusually A+T rich, when compared with the entire genome (Figure 6A). Moreover, the codon adaption index (CAI) indicates the absent/divergent genes have a low score and possess non-optimum codon usage (Figure 6B). Both of these measures suggest that many of the apparently absent/divergent genes identified in *S. lividans *strains have in fact been recently acquired by *S. coelicolor *and our results are consistent with the results of previous studies in this respect [4,30].

Our results are broadly consistent with the findings reported by Jayapal *et al *[30] in that the majority of genes absent in *S. lividans *66 and TK24 are present in the GI's and Gi's of TK21 defined previously. In some cases our results correlate exactly with previous results, particularly when considering the smaller genomic islets (Gi's). For example, *S. lividans *TK24 possesses blocks of absent genes which match Gi-3, (SCO2862-2871), Gi-4, (SCO3250-3270), Gi-8 (SCO4210-4218), Gi-9 (SCO4346-4350), Gi-11 (SCO4615-4631), Gi-13 (SCO5605-5620), Gi-15 (SCO5718-5735) and the Right End terminal deletion (SCO7827-7845) of *S. lividans *TK21 exactly. Figure 3B illustrates Gi-11 in *S. lividans *66. Similarly, *S. lividans *66 possesses blocks of absent genes which match Gi-3, Gi-5, Gi-6 (SCO3980-3998), Gi7 (SCO4060-4066) and Gi-8.

However, there are also significant differences between our results and those reported previously [30], and in many cases these correspond to regions where our results do not provide evidence for the complete absence of large blocks of genes. Instead, we are able to classify at least one gene, and in some cases many more, within most of the GI/Gi's identified previously [30] as being present, raising doubt as to the GI/Gi's boundaries and in some cases the very existence of a GI/Gi at all. For example, Gi-5 is reported as an absence of SCO3929-SCO3937 [30] whereas we find that in both *S. lividans *TK24 and 66 that SCO3936 is classed as present. Additionally, Gi-2 was reported to lack SCO2381-2384 whereas we find that SCO2381 is present in both TK24 and 66. Moreover, Gi-1 is defined as an absence of SCO0090-0099 [30] whereas in TK24 we find only SCO0090-0091 and SCO0098 are absent, and in *S. lividans *66 only a single gene SCO0098 is absent. Furthermore, out of the 147 missing genes (SCO6806-6953) defined as GI-5, we find that TK24 has 131 missing genes, but significantly *S. lividans *66 only has 43 missing. Hence, due to the methods employed in this study we have succeeded in identifying many single genes, and small blocks of genes, located in between genomic islands/islets as absent/divergent. For example, we assign three consecutive secreted protein genes (SCO5013-5015) and the isolated secreted protein gene SCO5995 as absent in both *S. lividans *66 and TK24. We also assign SCO3521-3522, encoding an integral membrane protein and a transcriptional regulator respectively, as absent in TK24 only.

**Figure 7:**
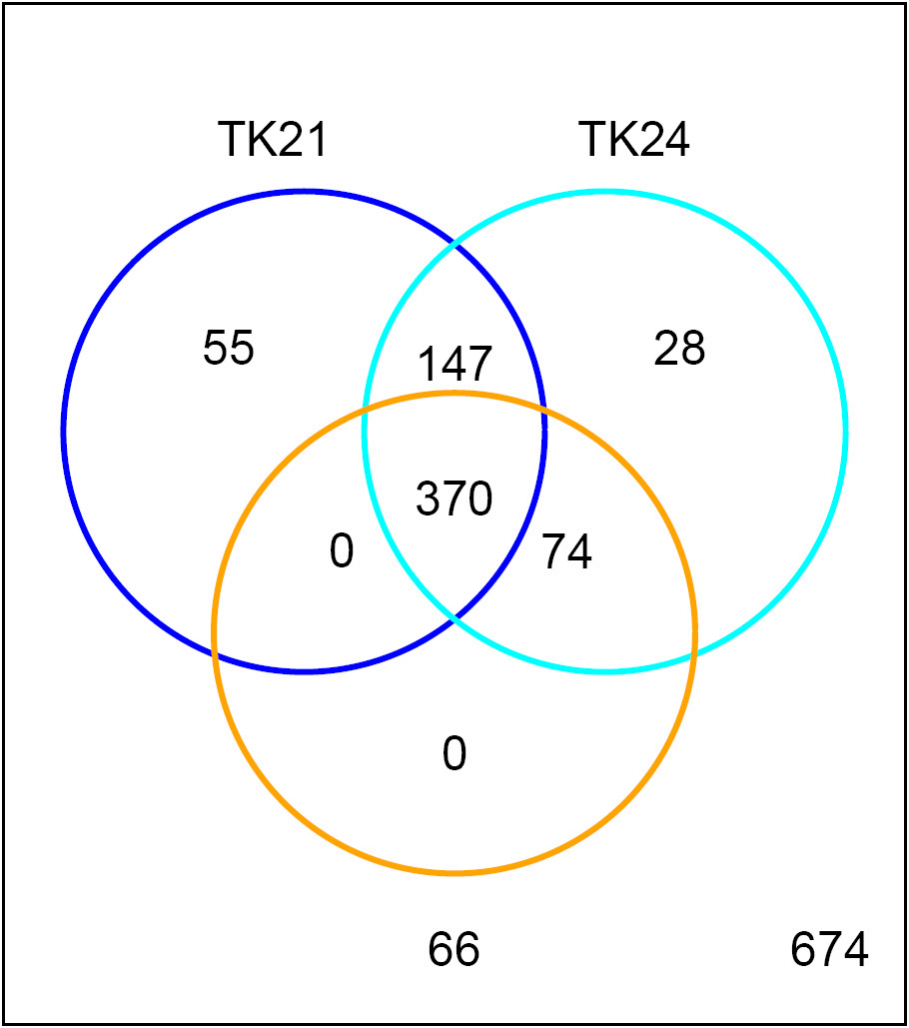
**Venn diagram of genes absent/divergent genes in *S. lividans *66, *S. lividans *TK24 and TK21, relative to *S. coelicolor *M145**.

It is interesting to note that the patterns of gene absence/divergence relative to *S. coelicolor *M145 differ in all three *S. lividans *strains so far investigated. The results are summarized in the Venn diagram shown in Figure 7 and indicate that whilst there appears to be a core of 370 absent genes common to all three strains there are also gene differences unique to one particular strain, or are shared between only two of them. This is consistent with the fact that although TK21 and TK24 were generated from *S. lividans *66 (John Innes Stock No. 1326) in the same study, they were generated in parallel [16]. It is likely that although some of the differences between TK21 and the other strains may be due to differences in the microarray platform employed and data analysis methodology used, these factors cannot account for the significant differences identified between *S. lividans *66 and TK24. It appears, *prima facie*, that TK21 and TK24 share more similar patterns of gene loss/divergence than do TK21 and 66, or TK24 and 66. TK24 and 66 have lost 74 genes relative to *S. coelicolor *M145 that were misclassified as present in TK21 from the previous lower resolution study [30]. Collectively, TK21 and TK24 lack 230 genes relative to the parent strain, *S. lividans *66 and some of these differences in gene loss might be attributed to the differences in plasmid profiles of the strains. CGH studies do not distinguish between chromosomal genes and those that are plasmid-located and can only inform as to their presence/absence. This being so it may be that loss of SLP2 from TK21 and TK24 explains some of the 147 absent genes common to these strains, which are present in *S. lividans *66, and the further loss of SLP3 by TK24 the additional 28 genes it has lost relative to the other strains. Furthermore, plasmids may have acquired chromosomal genes singly or in small blocks during repeated recombination events. It has long been known that SLP2 is able to mediate chromosomal recombination events [16] and that the rightmost 15.4 kb of SLP2 is identical to sequence from the *S. lividans *chromosome from which it is thought to have been recently acquired [39,40], probably by recombination occurring between the SLP2 and chromosomal copies of *Tn4811 *[41]. It has also been suggested that SLP3 is able to integrate into the *S. lividans *chromosome as curing of the plasmid has also resulted in deletion of chromosomal DNA [42].

### Consideration of the absent divergent genes in *S. lividans 66 *and TK24

The lists of genes which are absent/divergent and are common to *S. lividans *66 and TK24 (444) and are specific to TK24 (175) are listed in Additional Files 7, 8 and 9. Hypothetical genes and pseudogenes account for approximately 50% of the genetic differences between *S. lividans *66, *S. lividans *TK24, and *S. coelicolor *M145. It is clear from the gene annotations and co-localization pattern that a number of genes lost are from integrated plasmids of the pSAM2 family from *S. ambofaciens*. For example, genes SCO3250-3260 and SCO5336-5349 represent integrated plasmids similar to pSAM2 from which the three *S. lividans *strains have lost genes.

Other classes of genes well represented in the absent/divergent lists include transposases, and "orphan" membrane proteins, secreted proteins, transcription regulators, unspecified regulator and transporter proteins, lipoproteins, DNA-binding proteins, and ATP-binding proteins. Until such time as the precise functions of these genes are understood we are unable to explain or predict reasons for phenotypic differences between *S. coelicolor *and the *S. lividans *strains. Therefore, the main interest of the available datasets lies in the absent/divergent genes of *S. lividans *whose functional annotations are sufficiently detailed to be useful in hypothesis/prediction generation, *i.e*. mainly the enzymes.

The differences between the genetic complement of *S. lividans *TK21 and *S. coelicolor *M145 have been discussed elsewhere and we do not intend to reiterate the results of the previous analysis [30] and in view of their current annotations we are unable to usefully comment on the significance of the 28 absent/divergent genes specific to TK24. Of the 55 gene differences found in TK21 but not found in *S. lividans *66 and TK24, only three are enzymes and only one (SCO0984) possesses useful functional annotation, being a putative 3 hydroxyacyl-CoA dehydrogenase. However, TK21 also differs significantly from the other *S. lividans *strains in lacking the φC31 phage resistance gene *pglZ *(SCO6636), although it and TK24 both lack the φC31 phage resistance gene *pglY *(SCO6635). Both of these genes are present in *S. lividans *66.

### The differences in *act *biosynthesis between *S. lividans *and *S. coelicolor *may be due to absence/divergence of genes relating to S-adenosylmethionine

Among the more intriguing of the apparently missing genes in the *S. lividans *strains are those which are either involved in the biosynthesis of methionine or are S-adenosylmethionine (SAM)-dependent methyltransferases. The indirect involvement of genes possessing these functions in differentiation and antibiotic production has previously been noted [43]. Elevated concentrations of SAM have been shown to lead to expression of *actII-ORF4 *and subsequent overexpression of actinorhodin biosynthesis in *S. lividans *TK23 [44,45], overexpression of undecylprodiginine in *S. coelicolor *[46] and inhibition of sporulation in *S. lividans *[45]. SAM has been shown to mediate its effect on sporulation through induction of *bldK *expression [47], in addition to other ABC transporters involved in development [48], and possibly through modulation of *bldA *expression [46]. We note that the genes identified as missing in *S. lividans *66 and TK24 in the present study include SCO0985 (*metE*, encoding methionine synthase) and the methyltransferase genes SCO0995 (absent in both *S. lividans *66 and TK24) and SCO3452 (absent in TK24). It may be significant that both absent methyltransferase genes possess sequence similarity to SAM-dependent methyltransferases. Whilst we do not propose a direct and precise mechanism by which loss of SCO0985, SCO0995 and SCO3452 mediate the differences in antibiotic production and differentiation between *S. coelicolor *and *S. lividans *the roles we hypothesize for these genes and their effect on development and secondary metabolite biosynthesis relate to their effect on SAM concentration. This hypothesis is consistent with them playing roles similar to those outlined previously with regard to mutagenesis of the methionine synthase, *metH*, and a SAM dependent methyltransferase, SCO2525 [43].

### Investigation on sequence differences in *S.coelicolor *and *S. lividans bldB*

Although it is instructive to compare the macrosequence differences between the two *S. lividans *strains, and *S*. *coelicolor *much of the interest of the present dataset lies in the fine detail afforded by the high density coverage of the genomes, as exemplified above in the case of the CDA Asp-activating adenylation domains. A further example of the use of the dataset in microsequence analysis relates to the *bldB *gene (SCO5723). This gene encodes a small (98 aa) protein required for morphogenesis, antibiotic production and catabolite control in *S. coelicolor *[49]. *S. lividans *TK21 apparently lacks this gene [30] and in the present study we find it is classified as absent/divergent in *S. lividans *66 and TK24. A *bldB *homologue has long been known in *S. lividans *TK24 [50], its sequence has been determined (GenBank AF071232) and it is well known that *S. lividans *sporulates readily and does not exhibit the severe *bldB *deletant mutant phenotype observed in *S. coelicolor *mutants [51]. *bldB *null mutants have a "bald" phenotype when grown on all carbon sources and fail to produce aerial hyphae or antibiotics under any tested conditions [51,52]. In contrast to [30] we accept that *S. lividans *does in fact possess *bldB *and attribute its apparent absence in microarray studies to technical reasons relating to positioning of probe design and the pattern of sequence similarity between the *S. coelicolor *and *S. lividans *genes across their sequences (see Additional Files 10 &11). It seems that the PCR product used by Jayapal *et al *[30] spanning the entire TK21 *bldB *sequence was unable to bind to its target. In our microarrays the probes located 5" of *bldB *and the probe encompassing the 5" terminus (*i.e*. the start codon) were not bound in *S. lividans *66 or TK24, and of the three remaining probes directly, or partially targeting, *bldB *on our microarray three bound successfully to the 3' terminus of *bldB *in *S. lividans *66 and two bound successfully to TK24 genomic DNA (as determined by GACK analysis). Thus, in both *S. lividans *66 and TK24 the presence of three consecutive absent probes encompassing the translational start means that *bldB *is classed as absent/divergent when in fact it is present but possesses sequence sufficiently divergent from the *S. coelicolor *homologue in the 5" region to prevent hybridization. When the sequences of PCR products generated from both *S. lividans *strains using primers slbldBUp and slbldBDown (Additional File 1) were determined, both were found to be identical to the published *S. lividans bldB *sequence (GenBank: AF071232) and the TK24 *bldB *sequence identified by BLAST search of the Broad Institute database, showing that the *bldB *gene is indeed present in both *S. lividans *strains as well as in *S. coelicolor.*

### Investigation on metabolic differences between *S. coelicolor and S. lividans *and alternative mechanisms in *S. lividans *strains

Flux Balance Analysis (FBA) of Genome Scale Metabolic Reaction Networks (GSMN) has been widely used to predict metabolic capabilities of microbial strains, metabolic engineering of overproducing strains, prediction of essential genes and integration of high throughput data with the literature knowledge on metabolic reactions [53-55]. Here we have used FBA in combination with our CGH data to identify system-level differences in global metabolic flux distribution between *S. coelicolor *and *S. lividans*, and have modified the existing *S. coelicolor *GSMN accordingly and then tested our predictions relating to differences in cell physiology and C_2 _metabolism between the species.

The published GSMN model of *Streptomyces coelicolor *[54] was the starting point of our studies. The following nine metabolic genes which are included in the GSMN model were shown to be absent from the *S. lividans *66 genome by our CGH: SCO3486, SCO3479, SCO3473, SCO3494, SCO3474, SCO0982, SCO0983, SCO0985 & SCO6834. Subsequently, we identified all reactions, which require products of these genes and have removed them from the *S. coelicolor *GSMN model (Table 1). We checked whether the resulting model is feasible *i.e*. whether it reproduces the growth of *S. lividans *strains on a typical glucose-based minimal medium and then ran simulations of the maximal flux towards reactions representing biomass synthesis. This modified model, referred to here as the *S. lividans *GSMN, predicted that the metabolic network is capable of biomass synthesis.

**Table 1:** Reactions requiring products of genes missing from S. lividans but present in the S. coelicolor genome scale metabolic network.

ID	Reaction formula	E.C. Number	Genes
R60	LACTAL + NAD = LLAC + NADH	1.2.1.22	SCO3486
R71	LACTOSE = GLAC + GLC	3.2.1.23	SCO3479
R377	KDPG = PYR + G3P	4.1.2.14	SCO3473
R394	HYDROXYAKG = PYR + GLX	4.1.3.16	SCO3473
R379	KDG + ATP = KDPG + ADP	2.7.1.45	SCO3494
R381	KDG + ATP = KDPG + ADP	2.7.1.45	SCO3474
R386	ICIT = GLX + SUCC	4.1.3.1	SCO0982
R388	ACCOA + GLX = COA + MAL	2.3.3.9	SCO0983
R491	HCYS + MTHPTGLU = THPTGLU + MET	2.1.1.14	SCO0985
R576	OTHIOxt + NADPH = RTHIO + NAD	1.8.1.9	SCO6834

Reactions from the model of Borodina *et. al*. [54] requiring products of genes classified as missing in *S. lividans *66 and TK24 according to CGH data. Reaction IDs and metabolite names are set according to the model of [54]; full names of metabolites can be found in [70].

Perhaps the genes most fundamental to core metabolic activities which the present study identifies as absent in *S. lividans *are those which encode isocitrate lyase (SCO0982) and malate synthase (SCO0983), which are both involved in the glyoxylate bypass mechanism. This is a key system for the utilisation of compounds, such as fatty acids or acetate, which enter central metabolism at the level of acetyl-CoA. This mechanism involves the condensation of acetyl-CoA and oxaloacetate to form citrate, which is isomerized to isocitrate, which can in turn either be decarboxylated to form α-ketoglutarate in the TCA cycle or which can be cleaved by isocitrate lyase (ICL) to yield succinate and glyoxylate. Malate synthase (MS) is able to condense acetyl-CoA and glyoxylate to generate malate and CoA. Thus ICL and MS, together with enzymes from the Krebs Cycle, catalyse the net formation of succinyl-CoA from two molecules of acetyl-CoA. There is little published evidence for the expression of the glyoxylate bypass enzymes in *Streptomyces *species and it is well known that the glyoxylate bypass is not the sole mechanism by which C_2 _units may enter *Streptomyces *central metabolism [56,57]. Although *S. cinnamonensis *possesses ICL and MS genes extracts from cells grown in oil-based media have been shown not to possess ICL activity [56] and *S. collinus *has only been shown to possess ICL activity when grown in the presence of Tween, but not in acetate [57]. We note that an isoform of malate synthase (SCO6243) is present in both *S. lividans *66 and TK24 and that reaction R388 may be catalyzed by this enzyme. However, as both strains lack ICL the strains must necessarily be deficient in the glyoxylate bypass.

We have used the *S. coelicolor *and *S. lividans *models to make predictions about the growth of both species on medium comprising palmitate as the sole carbon source. Simulations of maximal biomass synthesis rate predicted that *S. coelicolor *is able, and *S. lividans *is not able, to use palmitate as a sole carbon source (maximal biomass synthesis rate of the *S. lividans *model was 0). This contradicted previously published experimental data indicating that *S. lividans *TK24 is capable of growth in media comprising triacylglycerides or oleic acid as sole carbon source [58,59] in addition to our own observations which demonstrate that *S*. *lividans *66 and TK24 are capable of growth in minimal media where the sole carbon sources are Tween 80 or palmitic acid (data not shown). The apparent non-essentiality of the glyoxylate bypass enzymes may be explained by the presence of an alternative metabolic pathway. A number of possible alternatives for C_2 _metabolism to the glyoxylate bypass mechanisms have been suggested, although they have not been fully characterized/confirmed [56,60]. However, the recently identified ethylmalonyl-CoA pathway [61,62] is the most likely candidate mechanism which operates as an alternative to the glyoxylate shunt in *S. lividans*; indeed, this pathway has recently been shown to operate in *S. coelicolor *(D. A. Hodgson, personal communication).

## Conclusions

The results presented here provide a powerful demonstration of the application of high-density microarrays to CGH studies. As shown here high-density IJISS arrays are capable of distinguishing between extremely similar sequences allowing specific discrimination on the basis of as few as three nucleotide mismatches.

The results presented here regarding single probe absences provide evidence for recombination between the aspartate-specific adenylation domain modules (4, 5 & 7) of the CdaPS genes of the CDA biosynthetic gene cluster revealing them to be "mosaic" genes, relative to the corresponding *S. coelicolor *sequences. We also identify an intergenic sequence divergence from *S. coelicolor *in the actinorhodin biosynthetic gene cluster and suggest that this, and/or the differences in the *S. lividans *complement of genes involved in SAM biosynthesis, or SAM-dependent methytransferases, may be involved in mediating the phenotypic differences in actinorhodin production between *S. coelicolor *and *S. lividans*.

Taking a broader view, the results indicate that the pattern of genetic differences between *S. lividans *66 and TK24 are different to those of *S. coelicolor *M145 and *S. lividans *TK21. We propose that more widespread genetic drift and recombination has occurred in *S. lividans *than the "archipelago model" developed previously, suggests (which focuses on "hotspots" of genetic difference).

In a series of case studies we confirm that the pattern of probe binding to *S. lividans *genes correlates with their sequence differences relative to *S. coelicolor *and use this to explain the published contradictory results previously reported for *S. lividans bldB*.

We have developed a GSMN for *S. lividans *taking into account the genetic differences relating to the differences in central metabolism between *S. lividans *66 and *S. coelicolor*, including the absence of genes encoding the enzymes of the glyoxylate bypass-isocitrate lyase and malate synthase. The fact that the GSMN indicated that both *S. lividans *strains should not be able to grow on fatty acids as sole carbon source, so contradicting the literature, suggests an alternative pathway to the glyoxylate shunt exists in *S. lividans *and it is likely that the hypothesized ethylmalonyl-CoA pathway fulfils this role.

It is clear from the case studies presented here regarding *bldB *and the genes which encode the glyoxylate bypass enzymes that the microarray data presented here has enormous potential to explain previously published observations and inform new hypotheses. We note that observations published in the previous study [30] regarding the putative identification of genes involved in DNA methylation systems of *S. lividans *have stimulated and informed further studies into this phenomenon [26]. We expect that the present, more extensive and detailed study will do likewise.

Finally, we envisage that these high-density arrays will find widespread application for rapid assessment of genome content in mutational studies of *S. coelicolor *and related species. It is well known that streptomycetes generally display high genetic instability and second-site mutations can arise frequently in the *S. coelicolor *genome when conducting targeted mutagenesis studies (our unpublished observations). The 104K array provides an efficient tool for comparing a new mutant with its immediate parent, to allow identification of mutants that have acquired additional, unwanted, deletions/duplications (which may be missed by 'next-generation' sequencing) and hence exclude them from further study.

## Methods

### Strains and culture conditions

Strains used were *S. lividans *66 SLP2^+ ^SLP3^+ ^corresponding to NRRL Code B-16637 and ICSSB Number 1023 (corresponding to stock number 1326 from the John Innes Centre collection) [16], *S. lividans *TK24 (*str-6*, SLP2^-^, SLP3^-^) [16] and *S. coelicolor *A3(2) strain M145 [28]. *S. coelicolor *and *S. lividans *cultures for genomic DNA preparation were grown in YEME plus 34% sucrose medium supplemented with 0.5% glycine and grown at 30°C for 72 h until early stationary phase. DNA was extracted using the Kirby mix procedure [28].

### Microarray Design

A *S. coelicolor *high-density IJISS microarray comprising almost 104,000 60-mer experimentally assessed probes was produced and validated for CGH in this study. The initial rounds of probe design have been described previously [32] where the authors reported the use of a 44,000 60-mer probe array; briefly, a large database of all possible 60-mer probes based on the *S. coelicolor *A3(2) M145 genome (EMBL accession AL645882 version 2) was developed and the best performing probes, validated in terms of hybridisation quality and reduction of signal with the introduction of mismatches, were selected to optimise the array sensitivity and specificity. For the present study 103,695 experimentally validated probes, were selected to cover both coding and non-coding genomic regions, with an average spacing of 30 nucleotides; the probes were distributed randomly on the microarray. The *S. coelicolor *104K microarrays are available from Oxford Gene Technology Ltd (UK).

### Microarray hybridization and processing

Labelling reactions were performed using the BioPrime kit (Invitrogen). DNA (0.1-1 μg) was denatured at 94°C for 3 min in 40 μl including 20 μl 2.5 × random primer mix and kept on ice. Nucleotide mix, 5 μl (2 mM dATP, 2 mM dGTP, 2 mM dTTP, 0.5 mM dCTP), 3.75 μl Cy3/Cy5-dCTP (Perkin Elmer) and 1.5 μl of Klenow fragment (1.5 units) were added and the reaction was incubated at 37°C overnight. The labelled DNA was purified using the Minielute PCR purification kit (Qiagen) and the incorporated Cy3/Cy5-dCTP was quantified with the NanoDrop ND-1000 spectrophotometer. Two different genomic DNA preparations for both *S. coelicolor *M145 and *S. lividans *66 and one preparation of *S. lividans *TK24 were each analysed in a 'dye-balanced' experimental design (to remove any dye bias). Labelled gDNA (50 pmol) of each pair of strains was hybridised to the 104K arrays in a buffer containing 1 M NaCl/50 mM MES, pH 7/20% formamide/1% Triton X-100 and rotated at 55°C over 60 h. Each glass slide contains 2 × 104K arrays and thus both Cy3/Cy5 dye orientations for each genomic DNA sample of each strain pair were hybridized on the same slide. The arrays were then washed with Wash 1 [6 × standard saline phosphate/EDTA (0.18 M NaCl/10 mM phosphate, pH 7.4/1 mM EDTA) (SSPE)/0.005% N-lauryl sarcosine) and Wash 2 (0.06% SSPE/0.18% polyethylene glycol 200), both for 5 min at room temperature. Hybridised arrays were scanned using an Agilent Technologies microarray scanner (5 μm resolution) and the resultant images analysed using Agilent Technologies Feature Extraction software (Version 9.1.3.1) with local background correction.

### Microarray data processing

All microarray data were imported into R (version 2.5, R, R Development Core Team) and processed using the Bioconductor package Limma [63-65] and checked for spatial effects, of which none were found. By assuming the null hypothesis that *S. lividans *strains 66 and TK24 are similar to *S. coelicolor *M145 the data was transformed to log_2 _*S. lividans*/*S. coelicolor *ratios and normalised using within-array loess normalisation followed by the between-array scale function; by normalising all arrays together the data (and thus *S. lividans *strains) become directly comparable due to the M145 genome being present on each array. Probes on each array were flagged as poor quality if the signals of both channels were classified as outliers in at least one of the binary variables (1 for bad, 0 for good) of the Feature Extraction software (Agilent Technologies, variables: gIsFeatNonUnifOL/rIsFeatNonUnifOL, gIsBGNonUnifOL/rIsBGNonUnifOL, gIsFeatPopnOL/rIsFeatPopnOL and gIsBGPopnOL/rIsBGPopnOL). Probes that had at least two dye balanced values for each *S. lividans *strain experiment (*i.e*. good signals in two of the four arrays for the 66 vs M145 experiment and both arrays for TK24 vs M145) were selected for further processing. Note, where probes had three (non dye-balanced) good quality signals for the 66 vs M145 experiment one of the two values for the over-represented dye was selected randomly. For large intergenic regions the 104K arrays include complementary probes targeting both DNA strands to detect the presence of non-coding RNAs, as all samples used in this study are DNA, able to hybridise to both complementary probes, these signals were averaged. In total 97,611 probes targeting unique DNA regions were used for genomotyping analysis. The microarray design and data are available from ArrayExpress (Accession numbers, respectively, A-MAXD-28 and E-MAXD-58).

### Genomotyping analysis

For each of the 97,611 probes that passed filtering, median log_2 _ratios (taking the dye swapping into consideration) across biological replicates were calculated, resulting in one value for *S. lividans *66/*S. coelicolor *M145 and one value for *S. lividans *TK24/*S. coelicolor *M145. The resultant data was converted into suitable data inputs (pcl format) for GACK (Charles Kim, Stanford University) and processed using the default settings. Binary outputs of 0 or 1 were obtained to denote probe absence, or presence, respectively. "Regions of difference" between *S. lividans *66 or TK24 and *S. coelicolor *M145 were classified by sets of at least three probes. Thus, one or more probes with a GACK value of 1 (presence) separate regions of difference. Once all regions of difference were found absent genes could subsequently be identified by their annotated translational start codon; if a gene's translational start coordinate resides within an identified region of absence then it too was deemed as missing/divergent.

### Reciprocal BLAST between *Streptomyces *genomes

*S. coelicolor *protein-encoding gene nucleotide sequences were obtained from the EMBL genome file (Accession No. EMBL:AL645882.2]) in FASTA format. Protein-encoding gene nucleotide sequences for *S*. *lividans *TK24 were obtained from the genes.fasta file downloaded from the BROAD institute [66] on 12/12/2009. Local BLAST nucleotide databases were created from the obtained FASTA files and each gene sequence was blasted against the other database (*i.e. S. coelicolor *genes against the *S*. *lividans *database and *vice versa*). A gene was classed as present if in each of the blast results it had a sequence match of greater than 60 nt in length (at least the length of the probes on the array) with greater than 60% sequence identity and an expected value less than 0.01. If these criteria were not met then the gene was classified as absent.

### Codon Adaptation Index

The Codon Adaptation Index (CAI) value, a measure of synonymous codon usage bias, for each *S*. *coelicolor *protein encoding gene was calculated using the CAI tool of EMBOSS [67] The nucleotide sequences from the EMBL genome file (accession EMBL: AL645882.2) in FASTA format and the 'background' (genome frequency) codon usage table from the codon usage database [68,69] were used as input to EMBOSS:cai for calculating bias using default settings.

### PCR Analysis

Primer pairs, each specific for a particular region of *S. coelicolor *and/or *S. lividans *DNA sequence (Additional File 1), were obtained from Eurofins-MWG-Operon and used in colony PCR. One independently obtained PCR product for each *S. coelicolor *MT1110 CdaPS Asp-specific adenylation domain (Modules 4, 5 & 7) and two independently obtained PCR products for the corresponding sequences of *S. lividans *66, were separated on a 1% agarose/Tris-Acetate-EDTA gel, the relevant bands excised and the DNA extracted using a Promega Wizard™ SV Gel & PCR Clean-up system. The purified DNA was treated with PNK (NEB) and blunt end-cloned into *Sma*I cut dephosphorylated pUC18 (Fermentas) using T4 DNA ligase (NEB). The ligation mix was transformed into competent *E. coli *JM109 and insert containing clones were identified by blue/white selection. Plasmid DNA was prepared from such clones using Promega Wizard Plus™ minipreps DNA purification system and supplied to Eurofins-MWG-Operon for BigDye™ (ABI) sequencing using M13 forward (-43) and reverse (-49) universal primers.

PCR products for *S. coelicolor *M145 and *S. lividans *66 and TK24 *bldB *were similarly obtained, and treated, to determine the sequences of the central portions of *bldB *in these organisms/strains.

PCR products for a region spanning SCO5082 and SCO5083 incorporating the intergenic region for *S. coelicolor *M145 and *S. lividans *66 were also similarly obtained, and treated, to determine their sequences.

All of the *S. lividans *66 and TK24 sequences obtained in the course of this study are identical to those already publicly available from the *S. lividans *TK24 genome sequence [37,66].

### Flux Balance Analysis

The genome scale metabolic reaction network of Borodina *et al *[54] has been used as an initial model in the investigation of the system-wide metabolic differences of *S. coelicolor *and *S. lividans*. The reactions which require products of the genes, which according to our CGH are missing in *S. lividans *were removed from the network. Subsequently, the model has been refined by incorporation of additional reactions, which have been experimentally demonstrated to operate in *Streptomyces*, but were omitted from the initial model. The feasibility of growth of different *in silico *strains on different media has been simulated by the calculation of the maximal biomass synthesis rate with Flux Balance Analysis. Details of FBA methodology are described in detail elsewhere [53]. Linear Programming calculations have been done with GLPK library run from our software, which has been previously used to model metabolic reaction networks of *M. tuberculosis *and *S. coelicolo*r. Models used in this work are available in SBML format from the ScoFBA website [70]. The ScoFBA server also allows interactive simulations of these models *via *a web-based interface to our software.

## Authors' contributions

RL, NA and GB conducted the experiments. EL conducted the microarray data analysis and whole genome BLAST analysis. RL led the biological interpretation of the microarray data. RL and NA conducted other DNA sequence analysis. EL, GB, CPS, VB and MH conducted the design and validation of the 104K array. AMK conducted the flux balance analysis and, with RAL, revised the *S. coelicolor *GSMN model for *S. lividans *to accommodate the findings from the CGH analysis. RL, EL, NA, AMK and CPS wrote the paper. All authors read and approved the final manuscript.

## Supplementary Material

Additional file 1 **Sequences of oligonucleotide primers used in the present study**. Click here for file

Additional file 2 **Alignments of sequences derived from *S. coelicolor *M145 and *S. lividans *66 Modules 4, 5 & 7 from CDAPSI & II**. Sequences from SCO3230 (CDAPSI) and SCO3231 (CDAPSII) modules 4, 5 and 7 obtained in the course of the present study of *S. coelicolor *M145 ("SC") corresponding to nucleotide coordinates 3556889-3557382, 3560009-3560502 and 3567844-3568334 (Accession No. EMBL: AL645882.2) aligned with the corresponding sequences from *S. lividans *66 ("SL"). Nucleotides specific to *S. coelicolor *M145 module 4 are in yellow, nucleotides specific to *S. coelicolor *M145 modules 4 & 5 are in orange, nucleotides specific to *S. coelicolor *M145 module 5 are in red, nucleotides specific to *S. coelicolor *M145 modules 5& 7 are in purple, nucleotides specific to *S. coelicolor *M145 module 7 are in blue and nucleotides specific to *S. coelicolor *M145 modules 7 & 4 are in green. Nucleotide changes where the *S. lividans *66 sequence diverges from all of the *S. coelicolor *M145 module 4, 5 and 7 sequences are shown in pink. Click here for file

Additional file 3 **Sequence diversity in the SCO5082-SCO5083 intergenic region of the *act *cluster**. Sequences of *S. coelicolor *M145 (lower) and *S. lividans *66 (upper) corresponding to nucleotide coordinates 5524009-5524068 present in the SCO5082-SCO5083 intergenic region of the *S. coelicolor *M145 actinorhodin biosynthetic cluster (Accession No. EMBL: AL645882.2). Click here for file

Additional file 4 **Sequence of *S. coelicolor *M145 SCO6832 and SCO6833 and intergenic region**. (nucleotide coordinates 7602829-7604947) (Accession No. EMBL: AL645882.2). The coding sequences are shaded orange and the respective microarray probe positions are indicated by differently coloured text. The respective start and stop codons of the two genes are underlined. Click here for file

Additional file 5 **Table detailing probes illustrated in Additional File **4. The same colour coding is used for each probe. Binding/non-binding (as *per *GACK analysis) to *S. lividans *66 and TK24 is indicated. Click here for file

Additional file 6 **Alignment of *S. coelicolor *SCO6833 & SCO6832 and *S. lividans *TK24 homologues identified by BLAST search**. Click here for file

Additional file 7 **Genes classified as absent/divergent from *S. lividans *66 relative to *S. coelicolor***. Click here for file

Additional file 8 **Genes classified as absent/divergent from *S. lividans *TK24 relative to *S. coelicolor***. Click here for file

Additional file 9 **Genes classified as absent/divergent from *S. lividans *TK24 only**. Click here for file

Additional file 10 **Double-stranded nucleotide sequence of *S. coelicolor *M145 *bldB *region**. Double-stranded nucleotide sequence of *S. coelicolor *M145 *bldB *region with microarray probe positions marked above or below their corresponding sequences (nucleotide co-ordinates 6243830-6244327)(Accession No. EMBL: AL645882.2). *bldB *is highlighted in a light orange box, and the *S. lividans *TK24 *bldB *coding sequence is italicized and aligned below the *S. coelicolor *sequence; identical bases are indicated by asterisks. The respective start and stop codons of the two orthologous genes are underlined. Click here for file

Additional file 11 **Microarray probes illustrated in **Additional File 10**: binding/non-binding (as *per *GACK analysis) to *bldB *regions of *S. lividans *66 and TK24**. Click here for file
