# Supplementary material for: Metabolic and evolutionary insights into the closely-related species Streptomyces coelicolor and Streptomyces lividans deduced from high-resolution comparative genomic hybridization
Source: BMC Genomics. 2010 Dec 1;11:682. doi: 10.1186/1471-2164-11-682 (PMC3017869; doi:10.1186/1471-2164-11-682)
Supplement: Additional file 1 — Sequences of oligonucleotide primers used in the present study. [file 1471-2164-11-682-S1.DOC]

**Additional File 1.** List of oligonucleotides and their sequences used in the present study

| **Primer name** | **Sequence (5′→3′)** |
| --- | --- |
| Mod4livUp1 | ACCCGTGGAGAGGTACACGG |
| Mod4livDown1 | CGACGCGTCGAGGGCCTCG |
| Mod5livUp1 | ACCGCCCGCTTCGTACGACA |
| Mod5livDown1 | GCCCGGTCGAACGCCCCC |
| Mod7livUp1 | CCGGCGGCTGCATACGGTC |
| Mod47livDown1 | GGACGTGTCCAGGGCACGG |
| scbldBup | CAGGTGCCGGACGAGGACGTCAA |
| scbldBdown | CGACGCCCCCGTCGCCCGGC |
| slbldBup | GAGGCCGAGGACAAGGAACTCCG |
| slbldBdown | CGCCCCCGGAGCGCTCCGCG |
| actdelL | CGCTCTAGACTGCTGACCGCCGCGACCGA |
| actdelR | CGCAAGCTTTGAAGAACACCAGTGCCACC |
