# Supplementary material for: Metabolic and evolutionary insights into the closely-related species Streptomyces coelicolor and Streptomyces lividans deduced from high-resolution comparative genomic hybridization
Source: BMC Genomics. 2010 Dec 1;11:682. doi: 10.1186/1471-2164-11-682 (PMC3017869; doi:10.1186/1471-2164-11-682)
Supplement: Additional file 2 — Alignments of sequences derived from S. coelicolor M145 and S. lividans 66 Modules 4, 5 & 7 from CDAPSI & II. Sequences from SCO3230 (CDAPSI) and SCO3231 (CDAPSII) modules 4, 5 and 7 obtained in the course of the present study of S. coelicolor M145 ("SC") corresponding to nucleotide coordinates 3556889-3557382, 3560009-3560502 and 3567844-3568334 (Accession No. EMBL: AL645882.2) aligned with the corresponding sequences from S. lividans 66 ("SL"). Nucleotides specific to S. coelicolor M145 module 4 are in yellow, nucleotides specific to S. coelicolor M145 modules 4 & 5 are in orange, nucleotides specific to S. coelicolor M145 module 5 are in red, nucleotides specific to S. coelicolor M145 modules 5& 7 are in purple, nucleotides specific to S. coelicolor M145 module 7 are in blue and nucleotides specific to S. coelicolor M145 modules 7 & 4 are in green. Nucleotide changes where the S. lividans 66 sequence diverges from all of the S. coelicolor M145 module 4, 5 and 7 sequences are shown in pink. [file 1471-2164-11-682-S2.DOC]

Additional File 2

Mod4SC GAAGCCCTCCACACCGACCACCTCGTCACCTGGCGGACCCAGCACCCCGGCGTTCAGATC

Mod4SL GAAGCCCTCCACACCGACCACCTCGCCACCTGGCGCACCCAGCACCCCCACGTCCAGATC

Mod7SC GAAGCCCTCCACACCGACCAGCTGACCGACTGGCGCACCCAGCACCCCGGCGCCCAGATC

Mod7SL GAAGCCCTCCACACCGACCAGCTGACCGACTGGCGCACCCAGCACCCCGGCGCCCAGATC

Mod5SC GAAGCCCTCCACACCGACCACCTCGCCACCTGGCGCACCCAGCACCCCGGCGCCCAGATC

Mod5SL GAAGCCCTCCACACCGACCACCTCGCCACCTGGCGGACCCAGCACCCCGGCGTTCAGATC

******************** ** * ****** ************ ** ******

Mod4SC ATCAACGCCTACGGACCCACCGAATCCACCGTCAACATCACCGACCACCACGTAGGCGAA

Mod4SL ATCAACGCCTACGGACCCACCGAATCCACCGTCAACATCACCGACCACCGCCT---CGAC

Mod7SC ATCAACGCCTACGGACCCACCGAATCCACCGTCAACATCACCGACCACCGCCT---CGAC

Mod7SL ATCAACGCCTACGGACCCACCGAATCCACCGTCAACATCACCGACCACCGCCT---CGAC

Mod5SC ATCAACGCCTACGGGCCCACCGAATCCACCGTCAACATCACCGACCACCACGTAAGCGAA

Mod5SL ATCAACGCCTACGGGCCCACCGAATCCACCGTCAACATCACCGACCACCGCCT---CGAC

************** ********************************** * * ***

Mod4SC GACACCCCTGACGGTCCCGTCCCCATCGGACGGCCCTTCGCCAACACCCAGGTCTACGTC

Mod4SL GGCACGGAAGAAGGCCCCGTCCCCATCGGACGGCCCTTCGCCAACACCCAGGTCTACGTC

Mod7SC GGCACGGAAGAAGGCCCCGTCCCCATCGGACGGCCCTTCGCCAACACCCAGGTCTACGTC

Mod7SL GGCACGGAAGAAGGCCCCGTCCCCATCGGACGGCCCTTCGCCAACACCCAGGTCTACGTC

Mod5SC GACACCCCTGACGGTCCCGTCCCCATCGGACGGCCCTTCGCCAACACCCAGGTCTACGTC

Mod5SL GGCACGGAAGAAGGTTCCGTCCCCATCGGACGGCCCTTCGCCAACACCCAGGTCTACGTC

* *** ** ** ********************************************

Mod4SC CTCGACTCGGCCCTGCGCCCCGTCGCCCCCGGCGTCACCGGAGAGCTGTACCTCGCCGGT

Mod4SL CTCGACTCAGCCCTGCGGCCCGTCGCCCCCGGCGTCACCGGAGAGCTGTACCTCGCCGGA

Mod7SC CTCGACTCAGCCCTGCGGCCCGTCGCCCCCGGCACCACCGGAGAGCTGTACCTCGCCGGA

Mod7SL CTCGACTCAGCCCTGCGGCCCGTCGCCCCCGGCACCACCGGAGAGCTGTACCTCGCCGGA

Mod5SC CTCGACTCAGCCCTGCGGCCCGTCGCCCCCGGCGTCACCGGAGAGCTGTACCTCGCCGGT

Mod5SL CTGGACTCGGCCCTGCGGCCGGTCGCCCCCGGCGTCACCGGAGAGCTGTACCTCGCCGGA

** ***** ******** ** ************ ************************

Mod4SC GAACAGCTCGCCCGCGGCTACCTCGGCCGCCCCGCGCTGACCGCCGAACGCTTCACCGCC

Mod4SL GAACAACTCGCCCGCGGCTACCTCGGCCGCCCCGCCCTCACCGCCGAACGCTTCACCGCC

Mod7SC GAACAACTCGCCCGCGGCTACCTCGGCCGCCCCGCCCTCACCGCCGAACGCTTCACCGCC

Mod7SL GAACAACTCGCCCGCGGCTACCTCGGCCGCCCCGCCCTCACCGCCGAACGCTTCACCGCC

Mod5SC GAACAACTCGCCCGCGGCTACCTCGGCCGCCCCGCCCTCACCGCCGAACGCTTCACCGCC

Mod5SL GAACAACTCGCCCGCGGCTACCTCGGCCGCTCCGCCCTCACCGCCGAACGCTTCACCGCC

***** ************************ **** ** *********************

Mod4SC AACCCCCACAGCAGCACCCCCGGCGCCCGCATGTACCGCACCGGTGACCTCGCCCACTGG

Mod4SL AACCCCCACAGCAGCACCCCCGGCGCCCGCATGTACCGCACCGGCGACCTCGCCCACTGG

Mod7SC AACCCCCACAGCAGCGTTCCCGGCGCCCGCATGTACCGCACCGGCGACCTCGCCCACTGG

Mod7SL AACCCCCACAGCAGCGTTCCCGGCGCCCGCATGTACCGCACCGGCGACCTCGCCCACTGG

Mod5SC AACCCCCACAGCAGCACCCCCGGCGCCCGCATGTACCGCACCGGCGACCTCGCCCACTGG

Mod5SL AACCCCCATAGCAGCGTTCCCGGCGCCCGCATGTACCGCACCGGCGACCTCGCCCACTGG

******** ****** ************************** ***************

Mod4SC AACCACCACGGCCACCTCACCTACGACGGACGCGCCGACCACCAGATCAAACTCCGCGGC

Mod4SL AACCACGACGGCCACCTCACCTACGACGGACGCGCCGACCACCAGATCAAACTCCGCGGC

Mod7SC AACCACCACGGCCACCTCACCTACGACGGACGCGCCGACCACCAGATCAAACTCCGCGGC

Mod7SL AACCACCACGGCCACCTCACCTACGACGGACGCGCCGACCACCAGATCAAACTCCGCGGC

Mod5SC AACCACGACGGCCACCTCACCTACGACGGACGCGCCGACCACCAGATCAAACTCCGCGGC

Mod5SL AACCACCACGGCCACCTCACCTACGACGGACGCGCCGACCACCAGATCAAACTCCGCGGC

****** *****************************************************

Mod4SC CACCGCATCGAACCCGGTGAGATCGAAGCCACCCTCACCGCACAGACCGGCATCACCCAA

Mod4SL CACCGCATCGAACCCGGCGAAATCGAAACCACCCTCACCGCACAGACCGGCATCACCCAA

Mod7SC CACCGCATCGAACCCGGCGAGATCGAAACCACCCTCACCGCACAGACCGGCATCACCCAA

Mod7SL CACCGCATCGAACCCGGCGAGATCGAAACCACCCTCACCGCACAGACCGGCATCACCCAA

Mod5SC CACCGCATCGAACCCGGCGAGATCGAAACCACCCTCACCGCACAGACCGGCATCACCCAA

Mod5SL CACCGCATCGAACCCGGCGAGATCGAAACCACCCTCACCGCACAGCCCGGCATCACCCAA

***************** ** ****** ***************** **************

Mod4SC GCCACCGTCCAACT

Mod4SL GCCACCGTCCAACT

Mod7SC GCCACCGTCCAACT

Mod7SL GCCACCGTCCAACT

Mod5SC ACCACGGTCCAACT

Mod5SL GCCACCATCCAACT

**** *******
