# Supplementary material for: Metabolic and evolutionary insights into the closely-related species Streptomyces coelicolor and Streptomyces lividans deduced from high-resolution comparative genomic hybridization
Source: BMC Genomics. 2010 Dec 1;11:682. doi: 10.1186/1471-2164-11-682 (PMC3017869; doi:10.1186/1471-2164-11-682)
Supplement: Additional file 3 — Sequence diversity in the SCO5082-SCO5083 intergenic region of the act cluster. Sequences of S. coelicolor M145 (lower) and S. lividans 66 (upper) corresponding to nucleotide coordinates 5524009-5524068 present in the SCO5082-SCO5083 intergenic region of the S. coelicolor M145 actinorhodin biosynthetic cluster (Accession No. EMBL: AL645882.2). [file 1471-2164-11-682-S3.PPT]

## Slide 1
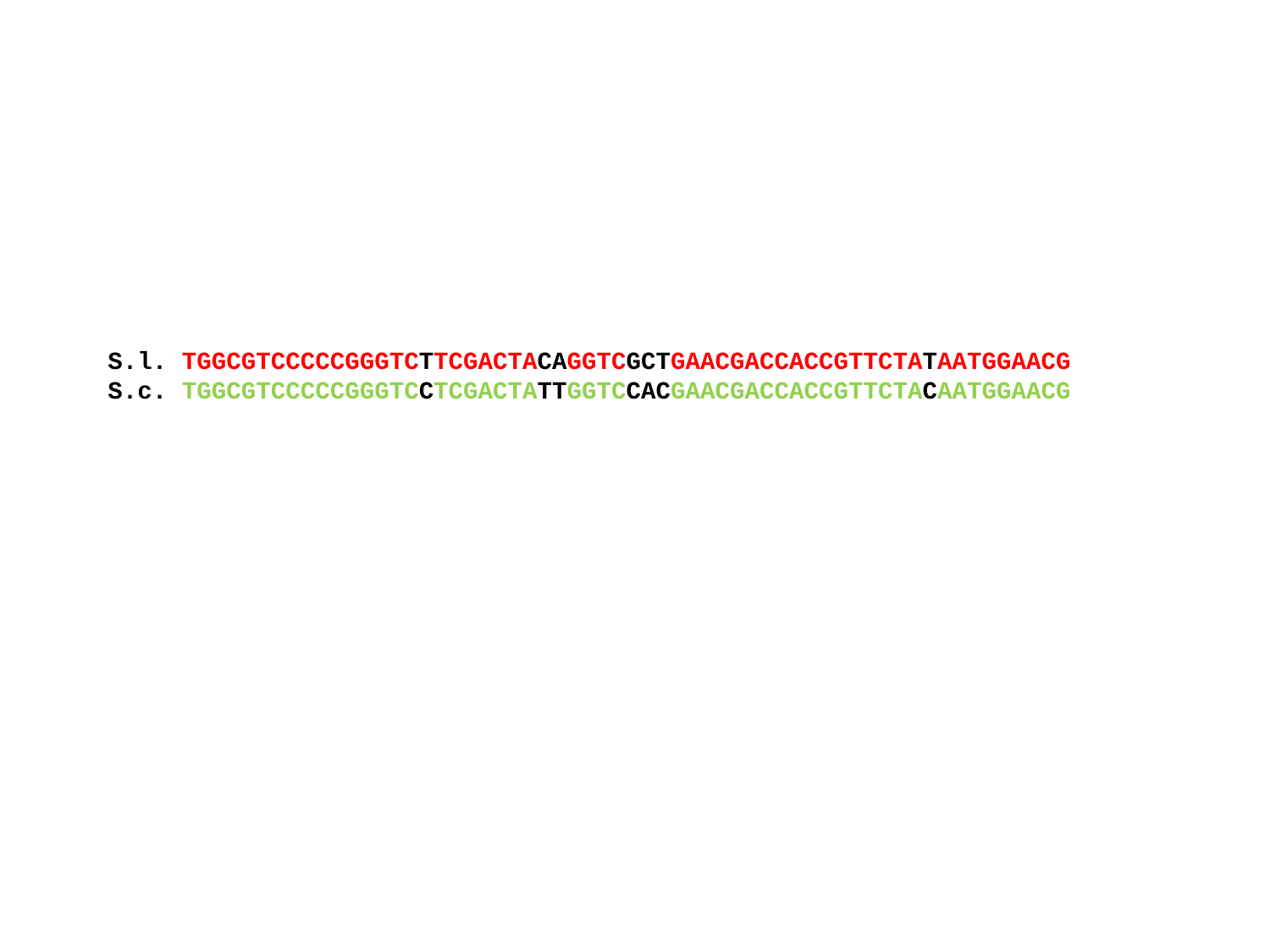

S.l. TGGCGTCCCCCGGGTCTTCGACTACAGGTCGCTGAACGACCACCGTTCTATAATGGAACG
S.c. TGGCGTCCCCCGGGTCCTCGACTATTGGTCCACGAACGACCACCGTTCTACAATGGAACG
