# Supplementary material for: Metabolic and evolutionary insights into the closely-related species Streptomyces coelicolor and Streptomyces lividans deduced from high-resolution comparative genomic hybridization
Source: BMC Genomics. 2010 Dec 1;11:682. doi: 10.1186/1471-2164-11-682 (PMC3017869; doi:10.1186/1471-2164-11-682)
Supplement: Additional file 4 — Sequence of S. coelicolor M145 SCO6832 and SCO6833 and intergenic region. (nucleotide coordinates 7602829-7604947) (Accession No. EMBL: AL645882.2). The coding sequences are shaded orange and the respective microarray probe positions are indicated by differently coloured text. The respective start and stop codons of the two genes are underlined. [file 1471-2164-11-682-S4.PPT]

## Slide 1
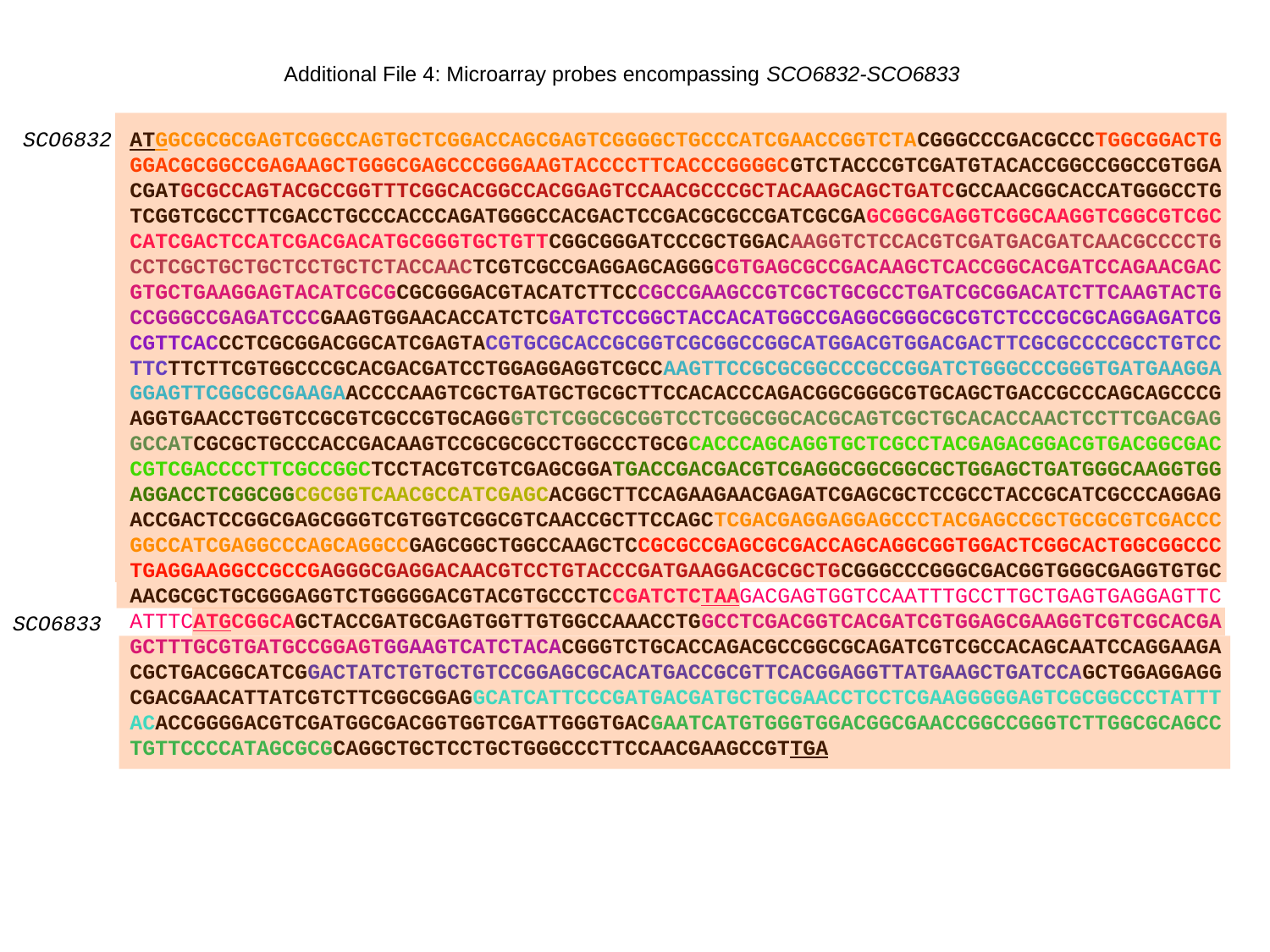

# Additional File 4: Microarray probes encompassing SCO6832-SCO6833
ATGGCGCGCGAGTCGGCCAGTGCTCGGACCAGCGAGTCGGGGCTGCCCATCGAACCGGTCTACGGGCCCGACGCCCTGGCGGACTGGGACGCGGCCGAGAAGCTGGGCGAGCCCGGGAAGTACCCCTTCACCCGGGGCGTCTACCCGTCGATGTACACCGGCCGGCCGTGGACGATGCGCCAGTACGCCGGTTTCGGCACGGCCACGGAGTCCAACGCCCGCTACAAGCAGCTGATCGCCAACGGCACCATGGGCCTGTCGGTCGCCTTCGACCTGCCCACCCAGATGGGCCACGACTCCGACGCGCCGATCGCGAGCGGCGAGGTCGGCAAGGTCGGCGTCGCCATCGACTCCATCGACGACATGCGGGTGCTGTTCGGCGGGATCCCGCTGGACAAGGTCTCCACGTCGATGACGATCAACGCCCCTGCCTCGCTGCTGCTCCTGCTCTACCAACTCGTCGCCGAGGAGCAGGGCGTGAGCGCCGACAAGCTCACCGGCACGATCCAGAACGACGTGCTGAAGGAGTACATCGCGCGCGGGACGTACATCTTCCCGCCGAAGCCGTCGCTGCGCCTGATCGCGGACATCTTCAAGTACTGCCGGGCCGAGATCCCGAAGTGGAACACCATCTCGATCTCCGGCTACCACATGGCCGAGGCGGGCGCGTCTCCCGCGCAGGAGATCGCGTTCACCCTCGCGGACGGCATCGAGTACGTGCGCACCGCGGTCGCGGCCGGCATGGACGTGGACGACTTCGCGCCCCGCCTGTCCTTCTTCTTCGTGGCCCGCACGACGATCCTGGAGGAGGTCGCCAAGTTCCGCGCGGCCCGCCGGATCTGGGCCCGGGTGATGAAGGAGGAGTTCGGCGCGAAGAACCCCAAGTCGCTGATGCTGCGCTTCCACACCCAGACGGCGGGCGTGCAGCTGACCGCCCAGCAGCCCGAGGTGAACCTGGTCCGCGTCGCCGTGCAGGGTCTCGGCGCGGTCCTCGGCGGCACGCAGTCGCTGCACACCAACTCCTTCGACGAGGCCATCGCGCTGCCCACCGACAAGTCCGCGCGCCTGGCCCTGCGCACCCAGCAGGTGCTCGCCTACGAGACGGACGTGACGGCGACCGTCGACCCCTTCGCCGGCTCCTACGTCGTCGAGCGGATGACCGACGACGTCGAGGCGGCGGCGCTGGAGCTGATGGGCAAGGTGGAGGACCTCGGCGGCGCGGTCAACGCCATCGAGCACGGCTTCCAGAAGAACGAGATCGAGCGCTCCGCCTACCGCATCGCCCAGGAGACCGACTCCGGCGAGCGGGTCGTGGTCGGCGTCAACCGCTTCCAGCTCGACGAGGAGGAGCCCTACGAGCCGCTGCGCGTCGACCCGGCCATCGAGGCCCAGCAGGCCGAGCGGCTGGCCAAGCTCCGCGCCGAGCGCGACCAGCAGGCGGTGGACTCGGCACTGGCGGCCCTGAGGAAGGCCGCCGAGGGCGAGGACAACGTCCTGTACCCGATGAAGGACGCGCTGCGGGCCCGGGCGACGGTGGGCGAGGTGTGCAACGCGCTGCGGGAGGTCTGGGGGACGTACGTGCCCTCCGATCTCTAAGACGAGTGGTCCAATTTGCCTTGCTGAGTGAGGAGTTCATTTCATGCGGCAGCTACCGATGCGAGTGGTTGTGGCCAAACCTGGCCTCGACGGTCACGATCGTGGAGCGAAGGTCGTCGCACGAGCTTTGCGTGATGCCGGAGTGGAAGTCATCTACACGGGTCTGCACCAGACGCCGGCGCAGATCGTCGCCACAGCAATCCAGGAAGACGCTGACGGCATCGGACTATCTGTGCTGTCCGGAGCGCACATGACCGCGTTCACGGAGGTTATGAAGCTGATCCAGCTGGAGGAGGCGACGAACATTATCGTCTTCGGCGGAGGCATCATTCCCGATGACGATGCTGCGAACCTCCTCGAAGGGGGAGTCGCGGCCCTATTTACACCGGGGACGTCGATGGCGACGGTGGTCGATTGGGTGACGAATCATGTGGGTGGACGGCGAACCGGCCGGGTCTTGGCGCAGCCTGTTCCCCATAGCGCGCAGGCTGCTCCTGCTGGGCCCTTCCAACGAAGCCGTTGA
SCO6832
SCO6833
