# Supplementary material for: Metabolic and evolutionary insights into the closely-related species Streptomyces coelicolor and Streptomyces lividans deduced from high-resolution comparative genomic hybridization
Source: BMC Genomics. 2010 Dec 1;11:682. doi: 10.1186/1471-2164-11-682 (PMC3017869; doi:10.1186/1471-2164-11-682)
Supplement: Additional file 5 — Table detailing probes illustrated in Additional File 4. The same colour coding is used for each probe. Binding/non-binding (as per GACK analysis) to S. lividans 66 and TK24 is indicated. [file 1471-2164-11-682-S5.DOC]

Additional File 5: Probe sequences encompassing SCO6832-SCO6833 – scored as present/absent from the CGH analysis. Colour coding corresponds to that used for each probe in Additional File 4.

| Probe sequence start nucleotide | Gene targeted | *S. lividans* 66  Presence (1)/ absence(0) | *S. lividans* TK24  Presence (1)/ absence |
| --- | --- | --- | --- |
| 7602830 | SCO6832 | 1 | 0 |
| 7602904 | SCO6832 | 1 | 1 |
| 7603004 | SCO6832 | 1 | 1 |
| 7603144 | SCO6832 | 1 | 1 |
| 7603224 | SCO6832 | 1 | 1 |
| 7603304 | SCO6832 | 1 | 1 |
| 7603384 | SCO6832 | 1 | 1 |
| 7603463 | SCO6832 | 1 | 1 |
| 7603544 | SCO6832 | 1 | 1 |
| 7603644 | SCO6832 | 1 | 1 |
| 7603804 | SCO6832 | 1 | 1 |
| 7603904 | SCO6832 | 1 | 1 |
| 7603984 | SCO6832 | 1 | 1 |
| 7604004 | SCO6832 | 1 | 1 |
| 7604164 | SCO6832 | 1 | 1 |
| 7604244 | SCO6832 | 1 | 1 |
| 7604284 | SCO6832 | 1 | 0 |
| 7604414 | SCO6832/SCO6833 | 0 | 0 |
| 7604507 | SCO6833 | 1 | 0 |
| 7604521 | SCO6833 | 1 | 0 |
| 7604648 | SCO6833 | 1 | 0 |
| 7604747 | SCO6833 | 0 | 0 |
| 7604847 | SCO6833 | 0 | 0 |
