# Supplementary material for: Metabolic and evolutionary insights into the closely-related species Streptomyces coelicolor and Streptomyces lividans deduced from high-resolution comparative genomic hybridization
Source: BMC Genomics. 2010 Dec 1;11:682. doi: 10.1186/1471-2164-11-682 (PMC3017869; doi:10.1186/1471-2164-11-682)
Supplement: Additional file 6 — Alignment of S. coelicolor SCO6833 & SCO6832 and S. lividans TK24 homologues identified by BLAST search. [file 1471-2164-11-682-S6.DOC]

Additional File 6

SCO6832 ATGGCGCGCGAGTCGGCCAGTGCTCGGACCAGCGAGTCGGGGCTGCCCATCGAACCGGTC 60

TK24_gene ----------------ATGACGCGCGAGTC--CGAGTCCGGGCTGCCCATCGAACCGGTC 42

** ** * ****** *********************

SCO6832 TACGGGCCCGACGCCCTGGCGGACTGGGACGCGGCCGAGAAGCTGGGCGAGCCCGGGAAG 120

TK24_gene TACGGGCCCGAAGCCCTGGCGGACTGGGAGGCGGCCGAGAAGCTGGGCGAGCCCGGGAAG 102

*********** ***************** ******************************

SCO6832 TACCCCTTCACCCGGGGCGTCTACCCGTCGATGTACACCGGCCGGCCGTGGACGATGCGC 180

TK24_gene TACCCGTTCACCCGGGGCGTGTACCCGTCCATGTACACCGGCCGGCCGTGGACGATGCGC 162

***** ************** ******** ******************************

SCO6832 CAGTACGCCGGTTTCGGCACGGCCACGGAGTCCAACGCCCGCTACAAGCAGCTGATCGCC 240

TK24_gene CAGTACGCCGGTTTCGGCACGGCGACGGAGTCCAACGCCCGCTACAAGCAGCTGATCGCC 222

*********************** ************************************

SCO6832 AACGGCACCATGGGCCTGTCGGTCGCCTTCGACCTGCCCACCCAGATGGGCCACGACTCC 300

TK24_gene AACGGCACCATGGGCCTGTCGGTCGCCTTCGACCTGCCCACCCAGATGGGCCACGACTCC 282

************************************************************

SCO6832 GACGCGCCGATCGCGAGCGGCGAGGTCGGCAAGGTCGGCGTCGCCATCGACTCCATCGAC 360

TK24_gene GACGCGCCGATCGCGAGCGGCGAGGTCGGCAAGGTCGGCGTCGCCATCGACTCCATCGAC 342

************************************************************

SCO6832 GACATGCGGGTGCTGTTCGGCGGGATCCCGCTGGACAAGGTCTCCACGTCGATGACGATC 420

TK24_gene GACATGCGCGTGCTGTTCGGCGGGATCCCGCTGGACAAGGTCTCCACGTCGATGACGATC 402

******** ***************************************************

SCO6832 AACGCCCCTGCCTCGCTGCTGCTCCTGCTCTACCAACTCGTCGCCGAGGAGCAGGGCGTG 480

TK24_gene AACGCCCCGGCCTCGCTGCTGCTCCTGCTCTACCAACTCGTCGCCGAGGAGCAGGGCGTG 462

******** ***************************************************

SCO6832 AGCGCCGACAAGCTCACCGGCACGATCCAGAACGACGTGCTGAAGGAGTACATCGCGCGC 540

TK24_gene AGCGCCGACAAGCTCACCGGCACGATCCAGAACGACGTGCTGAAGGAGTACATCGCGCGC 522

************************************************************

SCO6832 GGGACGTACATCTTCCCGCCGAAGCCGTCGCTGCGCCTGATCGCGGACATCTTCAAGTAC 600

TK24_gene GGGACGTACATCTTCCCGCCGAAGCCGTCGCTGCGCCTGATCGCGGACATCTTCAAGTAC 582

************************************************************

SCO6832 TGCCGGGCCGAGATCCCGAAGTGGAACACCATCTCGATCTCCGGCTACCACATGGCCGAG 660

TK24_gene TGCCGGGCCGAGATCCCGAAGTGGAACACCATCTCGATCTCCGGCTACCACATGGCCGAG 642

************************************************************

SCO6832 GCGGGCGCGTCTCCCGCGCAGGAGATCGCGTTCACCCTCGCGGACGGCATCGAGTACGTG 720

TK24_gene GCCGGTGCCTCGCCCGCGCAGGAGATCGCCTTCACCCTCGCGGACGGCATCGAGTACGTG 702

** ** ** ** ***************** ******************************

SCO6832 CGCACCGCGGTCGCGGCCGGCATGGACGTGGACGACTTCGCGCCCCGCCTGTCCTTCTTC 780

TK24_gene CGCACCGCGGTCGCGGCCGGCATGGACGTCGACGACTTCGCGCCCCGCCTCTCCTTCTTC 762

***************************** ******************** *********

SCO6832 TTCGTGGCCCGCACGACGATCCTGGAGGAGGTCGCCAAGTTCCGCGCGGCCCGCCGGATC 840

TK24_gene TTCGTGGCCCGCACGACGATCCTGGAGGAGGTCGCCAAGTTCCGCGCGGCCCGCCGGATC 822

************************************************************

SCO6832 TGGGCCCGGGTGATGAAGGAGGAGTTCGGCGCGAAGAACCCCAAGTCGCTGATGCTGCGC 900

TK24_gene TGGGCCCGGGTGATGAAGGAGGAGTTCGGCGCGAAGAACCCCAAGTCGCTGATGCTGCGC 882

************************************************************

SCO6832 TTCCACACCCAGACGGCGGGCGTGCAGCTGACCGCCCAGCAGCCCGAGGTGAACCTGGTC 960

TK24_gene TTCCACACCCAGACCGCGGGCGTGCAGCTGACCGCCCAGCAGCCCGAGGTGAACCTGGTG 942

************** ********************************************

SCO6832 CGCGTCGCCGTGCAGGGTCTCGGCGCGGTCCTCGGCGGCACGCAGTCGCTGCACACCAAC 1020

TK24_gene CGCGTCGCCGTGCAGGGCCTCGGCGCGGTCCTCGGCGGCACGCAGTCGCTGCACACCAAC 1002

***************** ******************************************

SCO6832 TCCTTCGACGAGGCCATCGCGCTGCCCACCGACAAGTCCGCGCGCCTGGCCCTGCGCACC 1080

TK24_gene TCCTTCGACGAGGCCATCGCGCTGCCCACCGACAAGTCCGCGCGCCTCGCCCTGCGCACC 1062

*********************************************** ************

SCO6832 CAGCAGGTGCTCGCCTACGAGACGGACGTGACGGCGACCGTCGACCCCTTCGCCGGCTCC 1140

TK24_gene CAGCAGGTGCTCGCCTACGAGACGGACGTGACGGCGACCGTCGACCCCTTCGCCGGCTCC 1122

************************************************************

SCO6832 TACGTCGTCGAGCGGATGACCGACGACGTCGAGGCGGCGGCGCTGGAGCTGATGGGCAAG 1200

TK24_gene TACGTCGTGGAGAAGATGACCGACGACGTCGAGGCGGCGGCGCTGGAGCTGATGGGCAAG 1182

******** *** **********************************************

SCO6832 GTGGAGGACCTCGGCGGCGCGGTCAACGCCATCGAGCACGGCTTCCAGAAGAACGAGATC 1260

TK24_gene GTCGAGGACCTCGGCGGCGCGGTCAACGCCATCGAGCACGGCTTCCAGAAGAGCGAGATC 1242

** ************************************************* *******

SCO6832 GAGCGCTCCGCCTACCGCATCGCCCAGGAGACCGACTCCGGCGAGCGGGTCGTGGTCGGC 1320

TK24_gene GAGCGCTCCGCCTACCGCATCGCCCAGGAGACCGACTCCGGCGAGCGGGTCGTGGTCGGC 1302

************************************************************

SCO6832 GTCAACCGCTTCCAGCTCGACGAGGAGGAGCCCTACGAGCCGCTGCGCGTCGACCCGGCC 1380

TK24_gene GTCAACCGCTACCAGCTCGACGAGGAGGAGCCCTACGAGCCGCTCCGCGTCGACCCGGCC 1362

********** ********************************* ***************

SCO6832 ATCGAGGCCCAGCAGGCCGAGCGGCTGGCCAAGCTCCGCGCCGAGCGCGACCAGCAGGCG 1440

TK24_gene ATCGAGGCCCAGCAGGCCGACCGGCTGGCCGCGCTCCGCGCGGAGCGCGACCAGACGGCG 1422

******************** ********* ********* ************ ****

SCO6832 GTGGACTCGGCACTGGCGGCCCTGAGGAAGGCCGCCGAGGGCGAGGACAACGTCCTGTAC 1500

TK24_gene GTGGACTCGGCCCTGGCGGCCCTGAAGAAGGCGGCCGAGGGCGAGGACAACGTCCTGTAC 1482

*********** ************* ****** ***************************

SCO6832 CCGATGAAGGACGCGCTGCGGGCCCGGGCGACGGTGGGCGAGGTGTGCAACGCGCTGCGG 1560

TK24_gene CCGATGAAGGACGCGCTGCGGGCGCGCGCGACGGTCGGCGAGGTGTGCAACGCGCTGCGG 1542

*********************** ** ******** ************************

SCO6832 GAGGTCTGGGGGACGTACGTGCCCTCCGAT---CTCTAA 1596

TK24_gene GAGGTCTGGGGGACCTACGTCCCTTCGGACGCGTTCTGA 1581

************** ***** ** ** ** *** *

SCO6833 ----ATGCGGCAGCTA--CCGATGCGAGTGGTTGTGGCCAAACCTGGCCTCGACGGTCAC 54

TK24_gene ATGGGTGTGGCAGCCGGTCCGATCCGCGTGGTGGTGGCCAAGCCGGGGCTCGACGGCCAC 60

** ****** ***** ** ***** ******** ** ** ******** ***

SCO6833 GATCGTGGAGCGAAGGTCGTCGCACGAGCTTTGCGTGATGCCGGAGTGGAAGTCATCTAC 114

TK24_gene GATCGCGGGGCCAAGGTGATCGCGAGGGCCCTGCGTGACGCCGGTATGGAGGTGATCTAC 120

***** ** ** ***** **** * ** ******* ***** **** ** ******

SCO6833 ACGGGTCTGCACCAGACGCCGGCGCAGATCGTCGCCACAGCAATCCAGGAAGACGCTGAC 174

TK24_gene ACCGGGCTCCACCAGACGCCCGAGCAGATCGTCGACACCGCGATCCAGGAGGACGCCGAC 180

** ** ** *********** * *********** *** ** ******** ***** ***

SCO6833 GGCATCGGACTATCTGTGCTGTCCGGAGCGCACATGACCGCGTTCACGGAGGTTATGAAG 234

TK24_gene GCGATCGGGCTGTCCATCCTCTCCGGTGCGCACAACACGCTCTTCGCCGCCGTGATCGAG 240

* ***** ** ** * ** ***** ******* ** *** * * ** ** **

SCO6833 CTGATCCAGCTGGAGGAGGCGACGAACATTATCGTCTTCGGCGGAGGCATCATTCCCGAT 294

TK24_gene CTGCTCCGGGAGCGGGACGCCGCGGACATCCTGGTCTTCGGCGGCGGGATCATCCCCGAG 300

*** *** * * *** ** ** **** * *********** ** ***** *****

SCO6833 GACGATGCTGCGAACCTCCTCGAAGGGGGAGTCGCGGCCCTATTTACACCGGGGACGTCG 354

TK24_gene GCGGACATCGCCCCGCTGAAGGAGAAGGGCGTCGCGGAGATCTTCACGCCCGGCGCCACC 360

* ** ** ** ** *** ******* * ** ** ** ** * *

SCO6833 ATGGCGACGGTGGTCGATTGGGTGACGAATCATGTGGGTGGACGGCGAACCGGCCGGGTC 414

TK24_gene ACGGCGTCCATCGTGGACTGGGT------CCGGGCGAACGTGCGG-GAGCCCGCGGGAGC 413

* **** * * ** ** ***** * * * * *** ** ** ** ** *

SCO6833 TTGGCGCAGCCTGTTCCCCATAGCGCGCAGGCTGCTCCTGCTGGGCCCTTCCAACGAAGC 474

TK24_gene ATAG-------------------------------------------------------- 417

* *

SCO6833 CGTTGA 480

TK24_gene ------
