# Supplementary material for: Metabolic and evolutionary insights into the closely-related species Streptomyces coelicolor and Streptomyces lividans deduced from high-resolution comparative genomic hybridization
Source: BMC Genomics. 2010 Dec 1;11:682. doi: 10.1186/1471-2164-11-682 (PMC3017869; doi:10.1186/1471-2164-11-682)
Supplement: Additional file 10 — Double-stranded nucleotide sequence of S. coelicolor M145 bldB region. Double-stranded nucleotide sequence of S. coelicolor M145 bldB region with microarray probe positions marked above or below their corresponding sequences (nucleotide co-ordinates 6243830-6244327)(Accession No. EMBL: AL645882.2). bldB is highlighted in a light orange box, and the S. lividans TK24 bldB coding sequence is italicized and aligned below the S. coelicolor sequence; identical bases are indicated by asterisks. The respective start and stop codons of the two orthologous genes are underlined. [file 1471-2164-11-682-S10.PPT]

## Slide 1
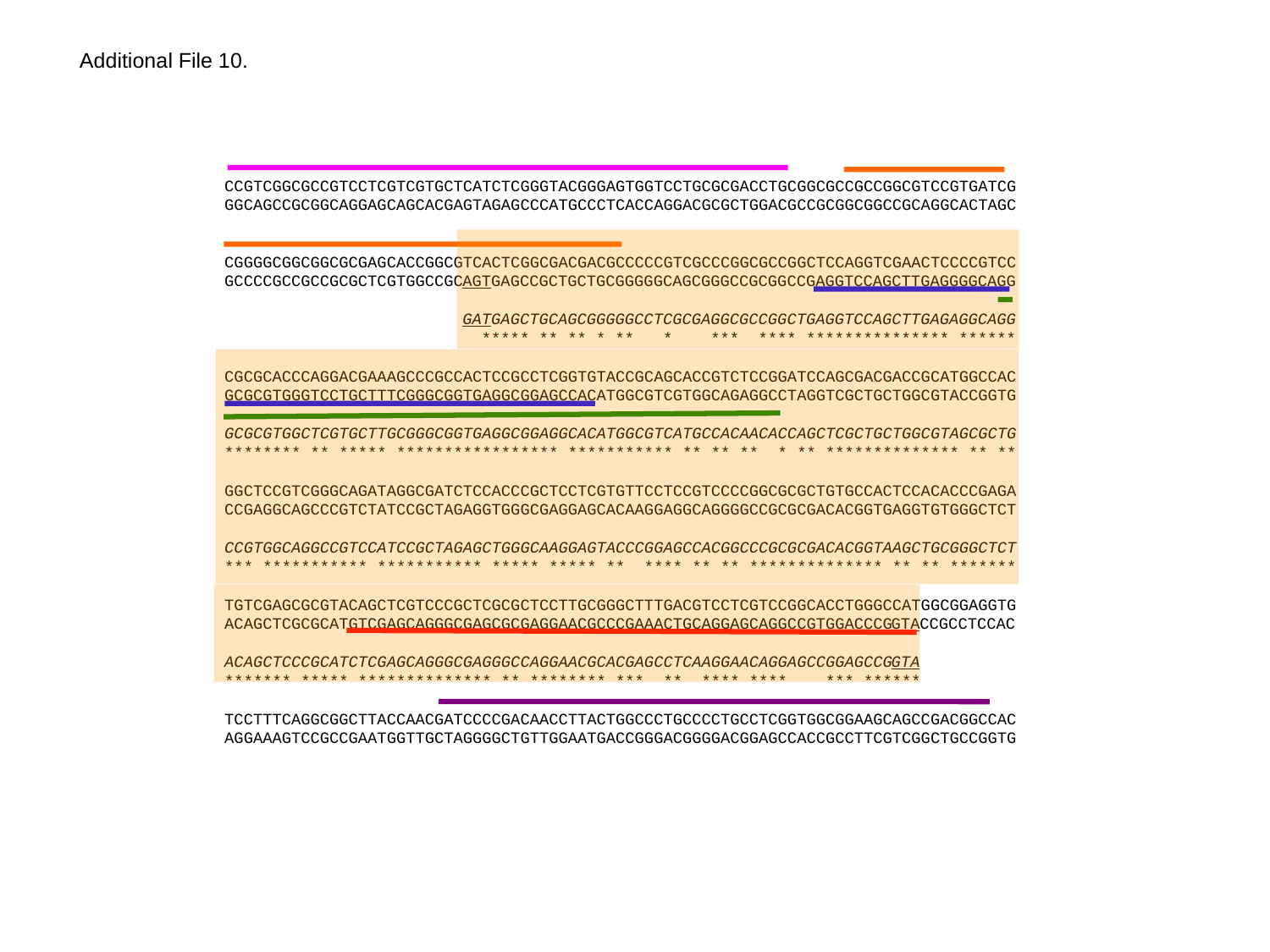

Additional File 10.
CCGTCGGCGCCGTCCTCGTCGTGCTCATCTCGGGTACGGGAGTGGTCCTGCGCGACCTGCGGCGCCGCCGGCGTCCGTGATCG
GGCAGCCGCGGCAGGAGCAGCACGAGTAGAGCCCATGCCCTCACCAGGACGCGCTGGACGCCGCGGCGGCCGCAGGCACTAGC
CGGGGCGGCGGCGCGAGCACCGGCGTCACTCGGCGACGACGCCCCCGTCGCCCGGCGCCGGCTCCAGGTCGAACTCCCCGTCC
GCCCCGCCGCCGCGCTCGTGGCCGCAGTGAGCCGCTGCTGCGGGGGCAGCGGGCCGCGGCCGAGGTCCAGCTTGAGGGGCAGG
 GATGAGCTGCAGCGGGGGCCTCGCGAGGCGCCGGCTGAGGTCCAGCTTGAGAGGCAGG
 ***** ** ** * ** * *** **** *************** ******
CGCGCACCCAGGACGAAAGCCCGCCACTCCGCCTCGGTGTACCGCAGCACCGTCTCCGGATCCAGCGACGACCGCATGGCCAC
GCGCGTGGGTCCTGCTTTCGGGCGGTGAGGCGGAGCCACATGGCGTCGTGGCAGAGGCCTAGGTCGCTGCTGGCGTACCGGTG
GCGCGTGGCTCGTGCTTGCGGGCGGTGAGGCGGAGGCACATGGCGTCATGCCACAACACCAGCTCGCTGCTGGCGTAGCGCTG
******** ** ***** ***************** *********** ** ** ** * ** ************** ** **
GGCTCCGTCGGGCAGATAGGCGATCTCCACCCGCTCCTCGTGTTCCTCCGTCCCCGGCGCGCTGTGCCACTCCACACCCGAGA
CCGAGGCAGCCCGTCTATCCGCTAGAGGTGGGCGAGGAGCACAAGGAGGCAGGGGCCGCGCGACACGGTGAGGTGTGGGCTCT
CCGTGGCAGGCCGTCCATCCGCTAGAGCTGGGCAAGGAGTACCCGGAGCCACGGCCCGCGCGACACGGTAAGCTGCGGGCTCT
*** *********** *********** ***** ***** ** **** ** ** ************** ** ** *******
TGTCGAGCGCGTACAGCTCGTCCCGCTCGCGCTCCTTGCGGGCTTTGACGTCCTCGTCCGGCACCTGGGCCATGGCGGAGGTG
ACAGCTCGCGCATGTCGAGCAGGGCGAGCGCGAGGAACGCCCGAAACTGCAGGAGCAGGCCGTGGACCCGGTACCGCCTCCAC
ACAGCTCCCGCATCTCGAGCAGGGCGAGGGCCAGGAACGCACGAGCCTCAAGGAACAGGAGCCGGAGCCGGTA
******* ***** ************** ** ******** *** ** **** **** *** ******
TCCTTTCAGGCGGCTTACCAACGATCCCCGACAACCTTACTGGCCCTGCCCCTGCCTCGGTGGCGGAAGCAGCCGACGGCCAC
AGGAAAGTCCGCCGAATGGTTGCTAGGGGCTGTTGGAATGACCGGGACGGGGACGGAGCCACCGCCTTCGTCGGCTGCCGGTG
