# Supplementary material for: Metabolic and evolutionary insights into the closely-related species Streptomyces coelicolor and Streptomyces lividans deduced from high-resolution comparative genomic hybridization
Source: BMC Genomics. 2010 Dec 1;11:682. doi: 10.1186/1471-2164-11-682 (PMC3017869; doi:10.1186/1471-2164-11-682)
Supplement: Additional file 11 — Microarray probes illustrated in Additional File 10: binding/non-binding (as per GACK analysis) to bldB regions of S. lividans 66 and TK24. [file 1471-2164-11-682-S11.DOC]

**Additional File 11**

| Probe sequence start nucleotide | *S. lividans* *66*  Presence (1)/ absence (0) | *S. lividans* TK24  Presence (1)/ absence (0) |
| --- | --- | --- |
| 6243829 | 0 | 0 |
| 6243894 | 1 | 1 |
| 6243974 | 1 | 1 |
| 6243994 | 1 | 0 |
| 6244174 | 0 | 0 |
| 6244266 | 0 | 0 |

Array probes, coloured as illustrated in Additional File 7A. Binding/non-binding (as *per* GACK analysis) to *S. lividans* *66* and TK24.
